# Phytochemistry and Pharmacological Potential of the Genus *Dalea*: Current Evidence and Future Directions

**DOI:** 10.3390/ph19081294

**Published:** 2026-08-15

**Authors:** Renata Solis-Peña, Vanessa Lizbeth Correa-Macias, Hania Lizeth Peralez-Olguin, Dalia Lizbeth Martínez-Medellín, Andrea Melissa González-Rodríguez, Lizeth Guadalupe Campos-Muzquiz, Lissethe Palomo-Ligas, Sendar Daniel Nery-Flores

**Affiliations:** Facultad de Ciencias Químicas, Universidad Autonoma de Coahuila, Unidad Saltillo, Saltillo 25280, Mexico; r-solis@uadec.edu.mx (R.S.-P.); vanessacorrea@uadec.edu.mx (V.L.C.-M.); haniaperalez@uadec.edu.mx (H.L.P.-O.); daliamedellin@uadec.edu.mx (D.L.M.-M.); gonzalez.melissa@uadec.edu.mx (A.M.G.-R.); l_campos@uadec.edu.mx (L.G.C.-M.); lissethe_palomo@uadec.edu.mx (L.P.-L.)

**Keywords:** natural products, flavonoids, prenylated metabolites, drug discovery, ethnopharmacology

## Abstract

The genus *Dalea* (Fabaceae) comprises approximately 188 recognized species distributed mainly in arid and semi-arid regions of the American continent, where several species have been traditionally used in folk medicine for the treatment of gastrointestinal disorders, infections, inflammation, and other ailments. In recent years, *Dalea* species have attracted considerable scientific interest due to their remarkable diversity of secondary metabolites, particularly prenylated flavonoids, isoflavonoids, rotenoids, chalcones, stilbenes, and terpenoid-rich essential oils. Among these compounds, prenylated and geranylated flavonoids represent some of the most characteristic metabolites of the genus and are strongly associated with its reported biological activities. This review summarizes current knowledge regarding the taxonomy, distribution, traditional uses, phytochemical profile, pharmacological activities, and toxicological aspects of the genus *Dalea*. Although only about 19 species (approximately 10% of currently recognized species) have been subjected to phytochemical investigation, substantial knowledge gaps remain within the genus. Special emphasis is placed on prenylated phenolic compounds because of their structural diversity and broad spectrum of biological activities, including antibacterial, antifungal, antiparasitic, anti-inflammatory, neuroprotective, anticancer, anti-tyrosinase, and xanthine-oxidase-inhibitory effects. Despite promising pharmacological findings, most available evidence remains limited to in vitro evaluations, with relatively few studies including in vivo validation or mechanistic investigations. Furthermore, important challenges remain regarding toxicity, pharmacokinetics, metabolomic characterization, and sustainable production of bioactive metabolites. Overall, the available evidence highlights *Dalea* as a valuable source of bioactive compounds with potential pharmaceutical applications and supports the need for further interdisciplinary research focused on drug discovery and development.

## 1. Introduction

Medicinal plants continue to play a pivotal role in the discovery and development of novel therapeutic agents, serving as a rich and diverse source of bioactive compounds with significant pharmacological potential [1]. It is estimated that a substantial proportion of the global population relies on traditional medicine, largely based on plant-derived preparations, for primary healthcare needs, with approximately 80% of people depending on traditional herbal medicine systems as their primary source of healthcare [2]. Beyond their ethnomedical relevance, medicinal plants have contributed directly to modern drug discovery, providing lead compounds or structural templates for the development of clinically relevant drugs [3]. The pharmacological potential of medicinal plants is primarily attributed to their secondary metabolites, including flavonoids, terpenoids, alkaloids, and phenolic acids [4]. These compounds exhibit a wide range of biological activities, such as antioxidant, anti-inflammatory, antimicrobial, and anticancer effects, often mediated through well-defined molecular targets and signaling pathways. In particular, increasing attention has been directed toward natural products capable of modulating key enzymes and pathways involved in oxidative stress, inflammation, and metabolic disorders, highlighting their potential as candidates for drug development [5,6,7].

In this context, species of the genus *Dalea* comprise a diverse group of leguminous plants distributed throughout the American continent, predominantly in arid and semi-arid regions [8]. Species within this genus exhibit a wide range of ecological adaptations that allow them to thrive under environmental stress conditions, which often leads to the production of specialized secondary metabolites with potential biological activity [9]. Although *Dalea* species have been primarily studied from a botanical and ecological perspective, increasing attention has been directed toward their phytochemical composition and pharmacological potential. Beyond their botanical interest, several *Dalea* species hold considerable ethnopharmacological value. Traditional medicinal uses have been documented among Indigenous communities across North and Central America, where these plants have been used to treat gastrointestinal disorders, inflammation, infections, respiratory conditions, and skin diseases [10,11]. For example, *Dalea carthagenensis* has been traditionally used in the Tehuacán-Cuicatlán Valley of Mexico to treat inflammation and gastrointestinal infections [12]. These traditional uses have motivated phytochemical and pharmacological studies that have identified bioactive compounds, particularly prenylated flavonoids, terpenoids, and phenolic compounds with antimicrobial, antioxidant, anti-inflammatory, cytotoxic, and enzyme-inhibitory activities [13,14,15].

Among the reported pharmacological activities, the inhibition of enzymes such as xanthine oxidase, acetylcholinesterase, and tyrosinase has attracted particular interest due to the potential therapeutic applications of these compounds in oxidative stress-related diseases, neurodegenerative disorders, and metabolic conditions [16,17,18]. Despite these promising findings, scientific information on *Dalea* species remains limited and fragmented, with most studies focusing on a small number of species and mainly in vitro evaluations. Therefore, the objective of this review is to compile and critically analyze the current knowledge on the ethnobotany, phytochemistry, pharmacological activities, and therapeutic potential of the *Dalea* genus, as well as to identify research gaps and future perspectives for drug discovery and pharmacological development.

## 2. Botanical Description and Phytogeography of *Dalea* Genus

### 2.1. Taxonomy and Classification

The genus *Dalea* belongs to the family Fabaceae (Leguminosae), subfamily Papilionoideae, and tribe Amorpheae. The subfamily Papilionoideae is one of the largest groups within Fabaceae, comprising approximately 14,000 species and more than 500 genera worldwide [19]. Within this subfamily, the tribe Amorpheae includes five genera (*Amorpha*, *Dalea*, *Eysenhardtia*, *Marina*, and *Psorothamnus*) and is primarily distributed in the Americas, where many of its species are adapted to arid and semi-arid environments [20]. The genus *Dalea* comprises approximately 188 recognized species distributed mainly throughout North, Central, and South America. The geographical distribution of *Dalea* extends from the southern United States to Chile, although the highest species diversity is found in Mexico. In South America, *Dalea* species are primarily distributed along the Andean region, from Colombia to Chile. Species of this genus are especially abundant in arid and semi-arid environments, where they constitute an important component of desert and xerophytic vegetation [21]. The genus *Dalea* was initially described by Linnaeus in 1737 and later revalidated and taxonomically redefined based on morphological characteristics, mainly by the number of stamens. Subsequent taxonomic revisions, especially those conducted by Barneby during the twentieth century, expanded the genus by incorporating species previously assigned to related genera such as *Psoralea*, *Petalostemon*, and *Kuhnistera*, thereby establishing its modern circumscription [22]. The taxonomic classification of the genus *Dalea* is summarized in Table 1.

### 2.2. Morphological Characteristics

Species within the genus *Dalea* are distinguished by a set of well-defined morphological traits. They typically exhibit an herbaceous, suffruticose, or shrub-like growth habit, ranging from small perennial herbs only a few centimeters tall to shrubs reaching approximately 2–3 m in height, depending on the species and habitat [22,24,25]. This variation in growth form reflects the broad ecological distribution of the genus across grasslands, deserts, and semi-arid ecosystems of the Americas. The stems and leaves bear punctate glands in ochre, yellow, or brown hues that often secrete aromatic oils. These glands are frequently present on the stems, rachis, leaflets, sepals, and occasionally on the petals. The leaves are predominantly imparipinnate, although trifoliolate arrangements may also occur, and the small leaflet size represents an adaptation to microphyllous vegetation typical of arid and semi-arid environments. The stipules are generally herbaceous and may show subglandular characteristics [8,26,27].

Diagnostic characters used for species identification within the genus include the presence and distribution of glands on stems, rachis, leaflets, and sepals; the number of leaflets; the position of foliar glands relative to the petiole; the presence of stipels; the indumentum of leaves, flowers, and fruits; the type of inflorescence; the size of the calyx tube relative to the total flower length; the presence of glands on the calyx; the differentiation of petals; and the morphology of the keel petal fusion. These morphological characteristics are essential for taxonomic identification and species delimitation within the genus [22,28]. The presence of glandular structures in several plant organs suggests the production and storage of secondary metabolites such as essential oils, terpenoids, and phenolic compounds, which may be associated with the biological activities reported for several *Dalea* species [29,30].

**Figure 1:**
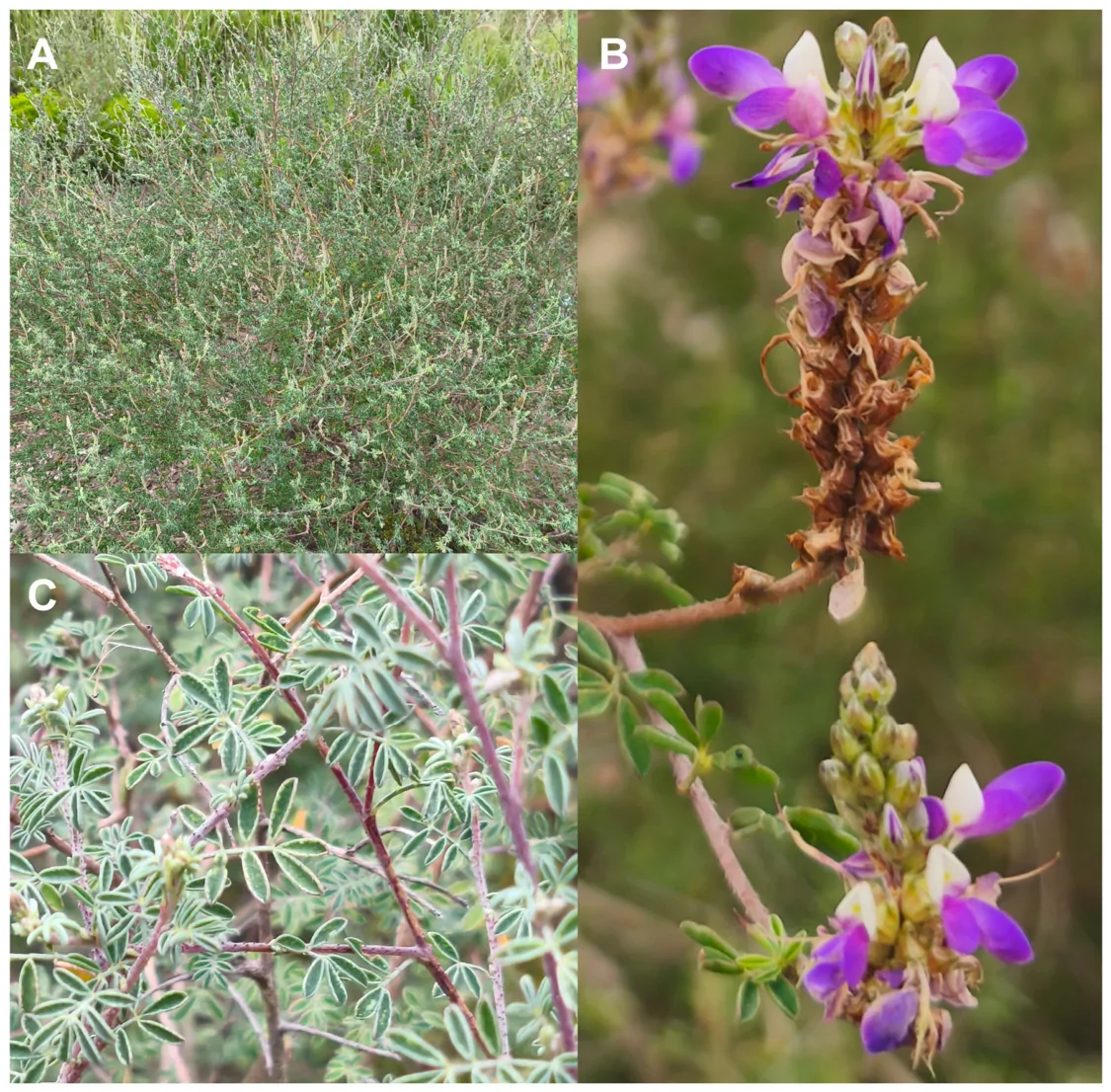
Morphological characteristics of *Dalea bicolor* in its natural habitat in northeastern Mexico. (**A**) General habit of the plant growing in semi-arid vegetation. (**B**) Spike-like inflorescences with papilionaceous purple flowers. (**C**) Detail of compound pinnate leaves with numerous small leaflets. Photographs by Peralez-Olguin and Nery-Flores.

The flowers of *Dalea* species are characterized by a tubular calyx with ten prominent ribs, which may be glabrous or pubescent, with five ribs extending into conspicuous teeth. The corolla is papilionaceous and consists of five petals differentiated into the standard (adaxial petal), wings (lateral petals), and keel (two fused lower petals), a typical feature of the Fabaceae family. The standard petal is commonly blue, purple, or pink, although in some species the flowers may initially open white or yellow and gradually darken with age [31]. The inflorescence architecture varies widely across the genus, representing one of the most distinctive morphological features of *Dalea*. Inflorescences range from dense, ovoid heads (e.g., *Dalea pulchra*) to elongated, conical spikes (e.g., *Dalea bicolor* var. *orcuttiana*) and cylindrical forms (e.g., *Dalea versicolor*) [9]. In *Dalea bicolor*, for instance, spike-like inflorescences bearing purple papilionaceous flowers can be clearly observed, along with compound pinnate leaves composed of numerous small leaflets (Figure 1). This morphological variability is an important taxonomic character for species identification within the genus. A notable example is *Dalea verticillata*, which is morphologically similar to *D. filiciformis* but can be distinguished by its verticillate phyllotaxy (typically five leaves per node), a higher number of leaflets, longer reflexed and persistent stipules, terminal paniculate inflorescences, and differences in petal morphology [21]. Floral morphology also plays a critical role in the taxonomic classification of the genus. Characters such as the shape of the keel petals, the fusion of petals, the length of the staminal column, and the morphology of epistemonous petals have been used to delimit sections and describe new species within *Dalea*, highlighting the taxonomic importance of floral structures in the genus [32].

### 2.3. Natural Habitat and Geographical Distribution

**Figure 2:**
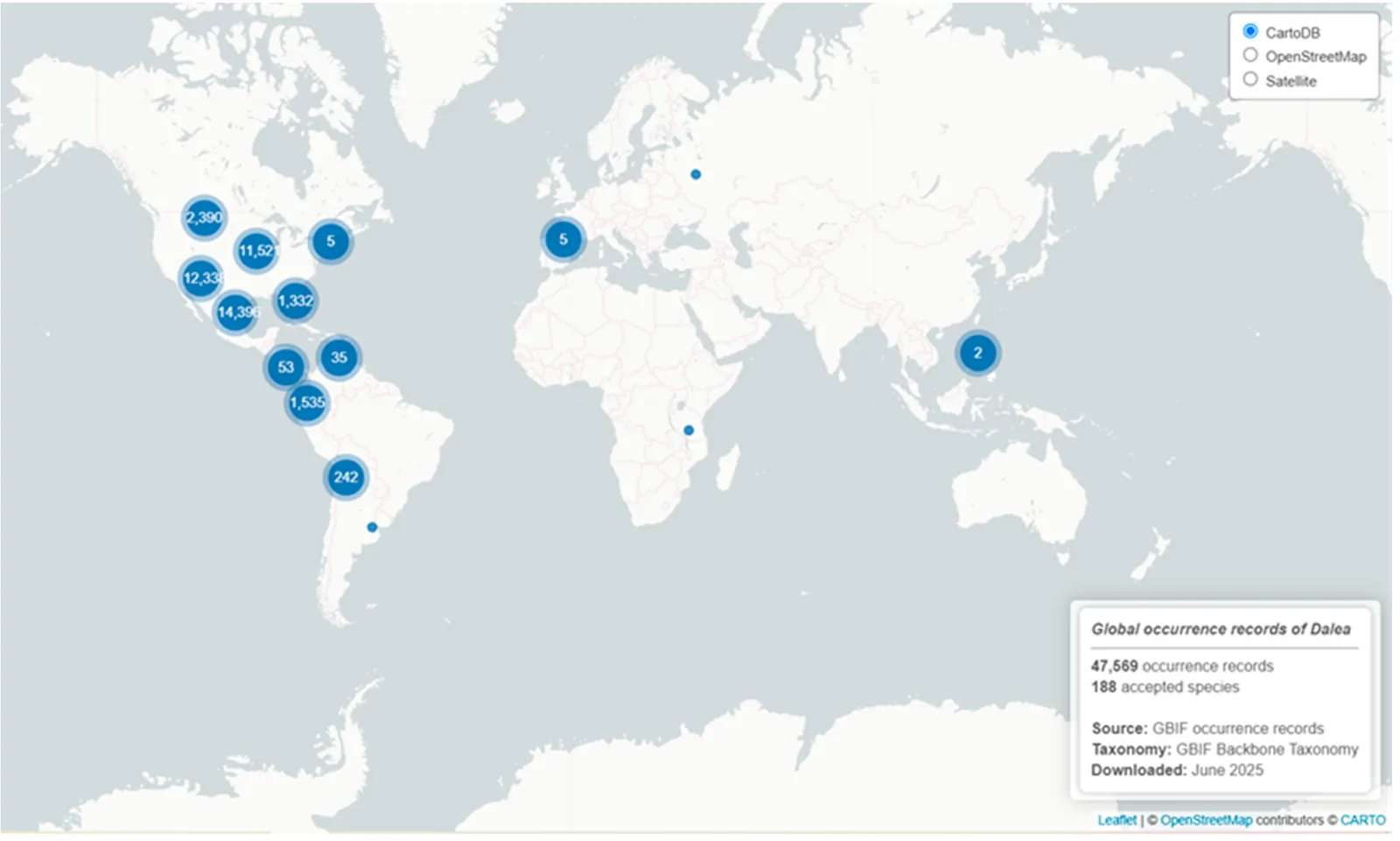
Interactive Leaflet map displaying global *Dalea* occurrence records retrieved from the Global Biodiversity Information Facility (GBIF.org, 2025) (https://doi.org/10.15468/dL.c6rbu3, accessed on 3 June 2025). Occurrence records are displayed as blue markers, while numbered circles represent clusters of nearby records that expand into individual occurrences as the user zooms in. Three selectable basemaps (CartoDB Positron, OpenStreetMap, and Esri World Imagery) are available in the interactive version at https://lizethmuzquiz.github.io/Dalea_GBIF_Resources/output/Dalea_interactive_map.html, accessed on 3 June 2025. The methodology, source code, and usage instructions are provided in Supplementary Material S1.

The genus *Dalea* is strongly associated with arid and semi-arid ecosystems of the American continent. One of the regions with the highest diversity of the genus is the Chihuahuan Desert, which hosts approximately 77 *Dalea* species, of which around 33 are endemic [27,33]. This highlights the ecological and evolutionary importance of this desert as a center of diversification for the genus. In these environments, *Dalea* species are commonly found in xerophytic shrublands, grasslands, sandy soils, rocky slopes, and disturbed habitats, where they play an important ecological role in desert plant communities [26]. In South America, *Dalea* species are mainly distributed along the Andean region, particularly in inter-Andean valleys and dry montane ecosystems, as well as tropical dry forests in some regions. Species of the genus can be found across a wide altitudinal gradient, ranging from lowland areas to elevations above 3000 m [15,22,26,34]. The harsh environmental conditions characterizing the natural habitats of *Dalea* species, including prolonged drought, intense solar radiation, nutrient-poor soils, and large daily temperature fluctuations, likely play an important role in shaping their phytochemical profiles. These abiotic stressors are widely recognized as drivers of plant secondary metabolism, promoting the biosynthesis and accumulation of protective metabolites such as flavonoids, terpenoids, and other phenolic compounds involved in antioxidant defense, UV protection, and adaptation to environmental stress [35,36,37]. Although direct experimental evidence remains scarce for *Dalea*, these ecological conditions are consistent with the broad diversity of specialized metabolites reported for the genus and suggest promising opportunities for future metabolomic studies aimed at understanding the influence of environmental factors on phytochemical variation. The remarkable ecological plasticity exhibited by several *Dalea* species further supports their adaptation to harsh environments. For instance, *Dalea cliffortiana* has been reported as naturalized in drought-exposed dryland habitats outside its native range, suggesting a high tolerance to water stress and other environmental constraints [38]. This broad ecological adaptability is reflected in the extensive geographic distribution of the genus across the Americas. The geographic distribution of the genus *Dalea* is shown in Figure 2. An interactive Leaflet map displaying global *Dalea* occurrence records generated from data retrieved from GBIF.org (2025), (https://doi.org/10.15468/dL.c6rbu3, accessed on 3 June 2025) together with the methodology used for its construction, access instructions, and the GitHub repository (https://github.com/lizethmuzquiz/Dalea_GBIF_Resources, accessed on 3 June 2025) containing the source code, are provided in Supplementary Material S1.

## 3. Phytochemical Profile of the Genus *Dalea*

### 3.1. Major Bioactive Compounds

**Figure 3:**
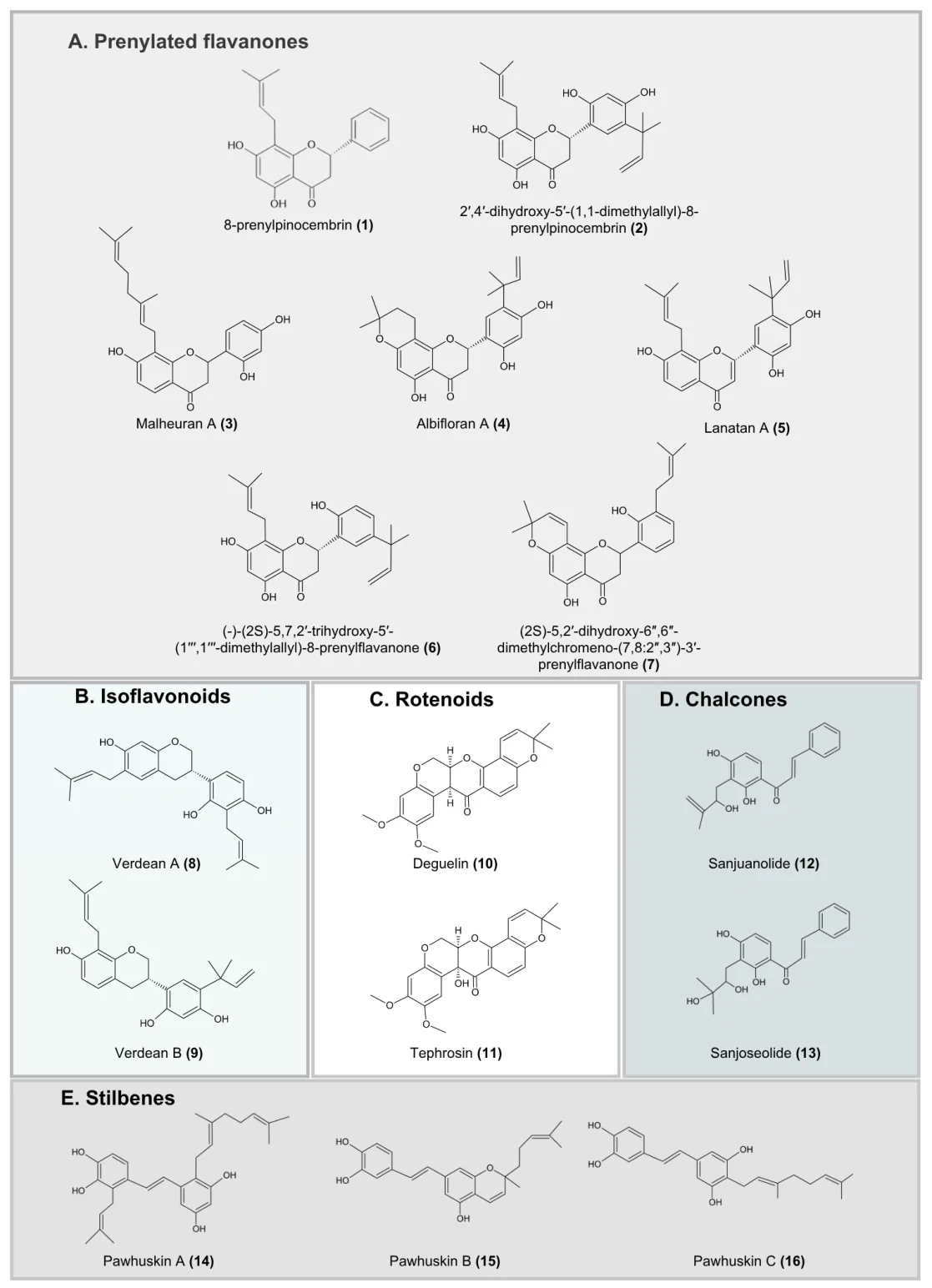
Representative non-volatile phytochemical classes reported in the genus *Dalea*. Species of the genus produce a wide diversity of phenolic secondary metabolites, including (**A**) prenylated flavonoids, (**B**) isoflavonoids, (**C**) rotenoids, (**D**) chalcones, and (**E**) stilbenes.

Species of the genus *Dalea* constitute an important source of secondary metabolites, particularly flavonoids, prenylated flavonoids, isoflavones, rotenoids, terpenoids, chalcones, pterocarpans, stilbenes and other biosynthetically related chemotype [39,40,41,42]. Many of these compounds have attracted scientific interest due to their diverse biological activities, including antimicrobial, anti-inflammatory, antioxidant, cytotoxic, and enzyme-inhibitory effects. Phytochemical investigations of different *Dalea* species have revealed a diverse of phenolic and terpenoid compounds distributed in roots, leaves, stems, flowers, and essential oils. These metabolites are considered responsible for many of the pharmacological properties attributed to the genus [43]. Available phytochemical evidence suggests a tissue-dependent distribution of specialized metabolites within the genus *Dalea*. Although prenylated flavonoids and other phenolic compounds have been isolated from both root and aerial tissues, the highest structural diversity of prenylated flavonoids, isoflavonoids, rotenoids, and stilbenes has been reported from roots. In contrast, studies of leaves, stems, and flowers have predominantly described volatile mono- and sesquiterpenes together with diverse phenolic constituents, including flavonoids and phenolic acids [10,43]. Among the phytochemical classes identified in *Dalea*, prenylated phenolic compounds constitute the most characteristic and pharmacologically attractive chemical scaffold reported to date. Their remarkable structural diversity, together with their broad spectrum of biological activities, makes them attractive lead structures for future drug discovery. The major phytochemical classes identified in species of the genus *Dalea*, together with representative metabolites, are summarized in Figure 3.

#### 3.1.1. Flavonoids

Flavonoids are among the most widely distributed secondary metabolites in the genus *Dalea* and represent one of the main classes of phenolic compounds reported in several species [9,30]. These compounds are typically found in leaves, stems, roots, flowers, and seeds, and include flavones, flavanones, isoflavones, flavonols, anthocyanin, chalcones and their glycosylated derivatives [44,45]. Flavonoids play important roles in plant physiology, including protection against ultraviolet radiation, temperature, oxidative stress, pathogens, and herbivores [46]. In the genus *Dalea*, flavonoids have been identified in multiple species and are considered responsible for many of the biological activities reported for the genus [17]. Several flavonoids isolated from *Dalea* species have shown antimicrobial, antioxidant, anti-inflammatory, and cytotoxic activities, suggesting their potential pharmacological relevance. The presence of flavonoids is also associated with the adaptation of *Dalea* species to arid and semi-arid environments, as these compounds contribute to plant adaptation in protection against oxidative stress and high solar radiation [47,48]. This ecological function may explain the high diversity of phenolic compounds reported in the genus. Regarding the flavonoids identified in *Dalea*, prenylated and geranylated derivatives represent one of the most distinctive and pharmacologically noteworthy groups of specialized metabolites. In plants, flavonoids may undergo structural modifications such as prenylation, geranylation, and farnesylation, involving the attachment of prenyl (C5), geranyl (C10), and farnesyl (C15) moieties to the flavonoid backbone [49,50]. Their occurrence in *Dalea* contributes substantially to the chemical identity of the genus and provides the basis for the diversity of bioactive phenolic compounds discussed in the following section.

#### 3.1.2. Prenylated Flavonoids

**Figure 4:**
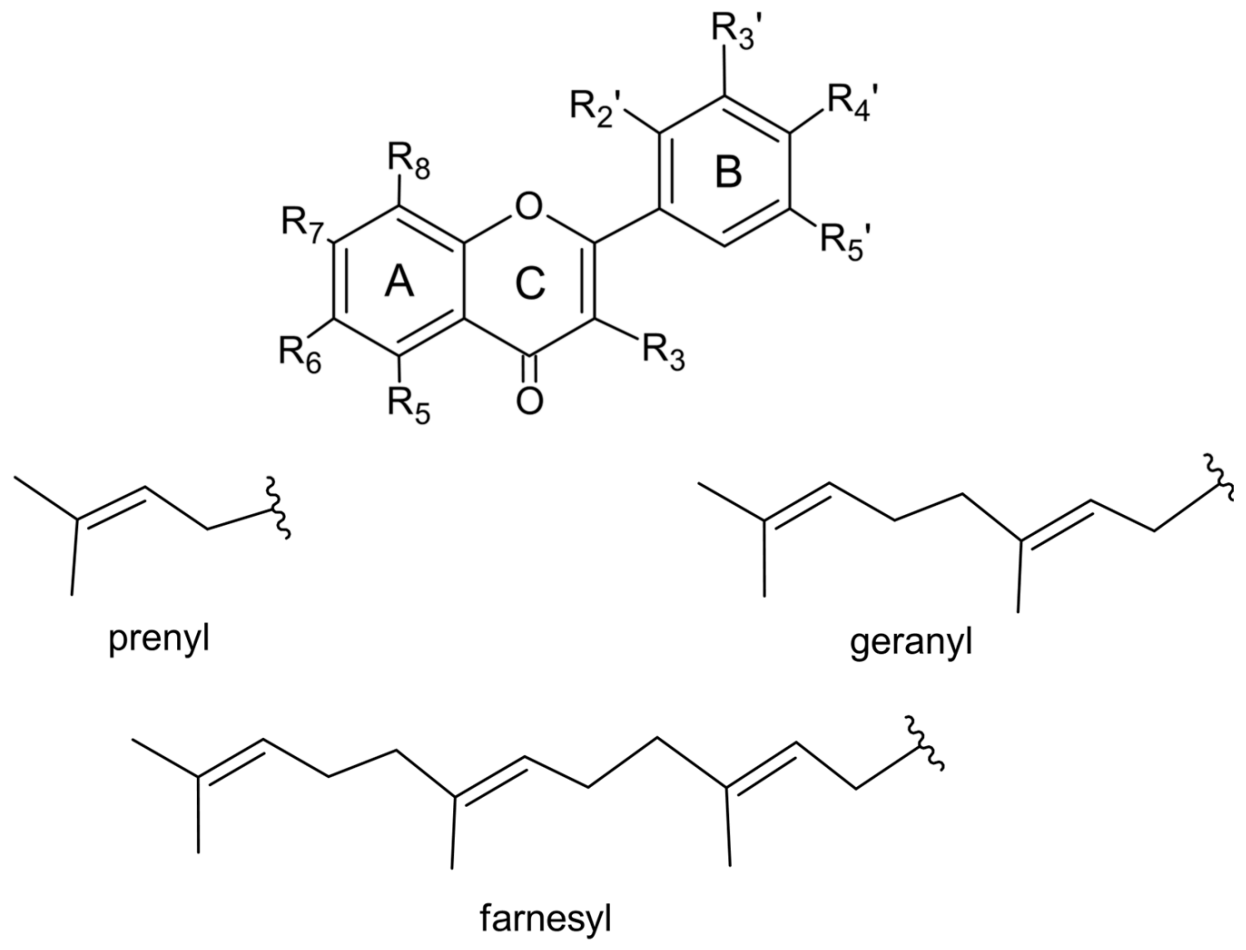
Structural modification of flavonoids through prenylation, geranylation, and farnesylation. Prenylated flavonoids are formed by the attachment of isoprenoid units such as prenyl (C5), geranyl (C10), and farnesyl (C15) groups to different positions of the flavonoid skeleton, commonly at C-6, C-8, C-3′, and C-5′. These modifications increase lipophilicity and significantly influence the biological activity of flavonoids.

Prenylated flavonoids constitute one of the most characteristic and pharmacologically relevant groups of specialized metabolites identified in the genus *Dalea*. Over the last decade, these compounds have emerged as because the introduction of prenyl or geranyl moieties generally enhances lipophilicity, membrane permeability, bioavailability, and affinity for biological targets compared with their non-prenylated counterparts, thereby increasing their therapeutic potential [13]. A recent comprehensive survey identified more than 1000 naturally occurring prenylated flavonoids distributed across 62 genera and 26 plant families, with Fabaceae and Moraceae representing the richest sources of these metabolites. Within these families, genera such as *Sophora*, *Glycyrrhiza*, *Erythrina*, *Morus*, and *Artocarpus* have been investigated extensively [51,52]. In comparison, although the genus *Dalea* has yielded a structural diversity of prenylated and geranylated flavonoids, it remains comparatively underexplored, highlighting its potential as a valuable source of chemically unique prenylated phenolic scaffolds for future phytochemical and pharmacological investigations. Prenylation is a common structural modification of plant secondary metabolites that involves the enzymatic attachment of active isoprenoid units called dimethylallyl diphosphate (DMAPP) into aromatic compounds such as flavonoids, coumarins, and phenolic acids [51]. In flavonoids, prenylation typically occurs through the addition of prenyl (C5), geranyl (C10), or farnesyl (C15) groups derived from isoprenoid biosynthesis pathways. This modification is catalyzed by prenyltransferase enzymes and represents an important diversification mechanism in plant secondary metabolism [53]. Prenyltransferases catalyze the regioselective transfer of isoprenoid units to specific positions of the flavonoid nucleus, most commonly at C-6 and C-8 of ring A, thereby generating the characteristic prenylated scaffolds reported in the genus *Dalea* [54]. Prenyl groups are usually attached to the flavonoid skeleton through carbon–carbon bonds (C-prenylation), although oxygen-linked prenylation (O-prenylation) may also occur. The most common prenylation positions in flavonoids are C-6 and C-8 on ring A, and occasionally C-3′ or C-5′ on ring B (Figure 4) [55,56].

Among the prenylated flavonoids reported in the genus *Dalea*, prenylated flavanones constitute the predominant and best-characterized subclass. Most of these compounds are structurally related to pinocembrin and its prenylated or geranylated derivatives, which have been isolated from species such as *D. elegans*, *D. boliviana*, *D. pazensis*, *D. searlsiae*, and more recently *D. albiflora* and *D. lanata*. Although prenylated isoflavonoids, chalcones, and stilbenes have also been identified, prenylated flavanones account for the majority of the bioactive metabolites reported to date and have been the focus of most pharmacological investigations.

**Figure 5:**
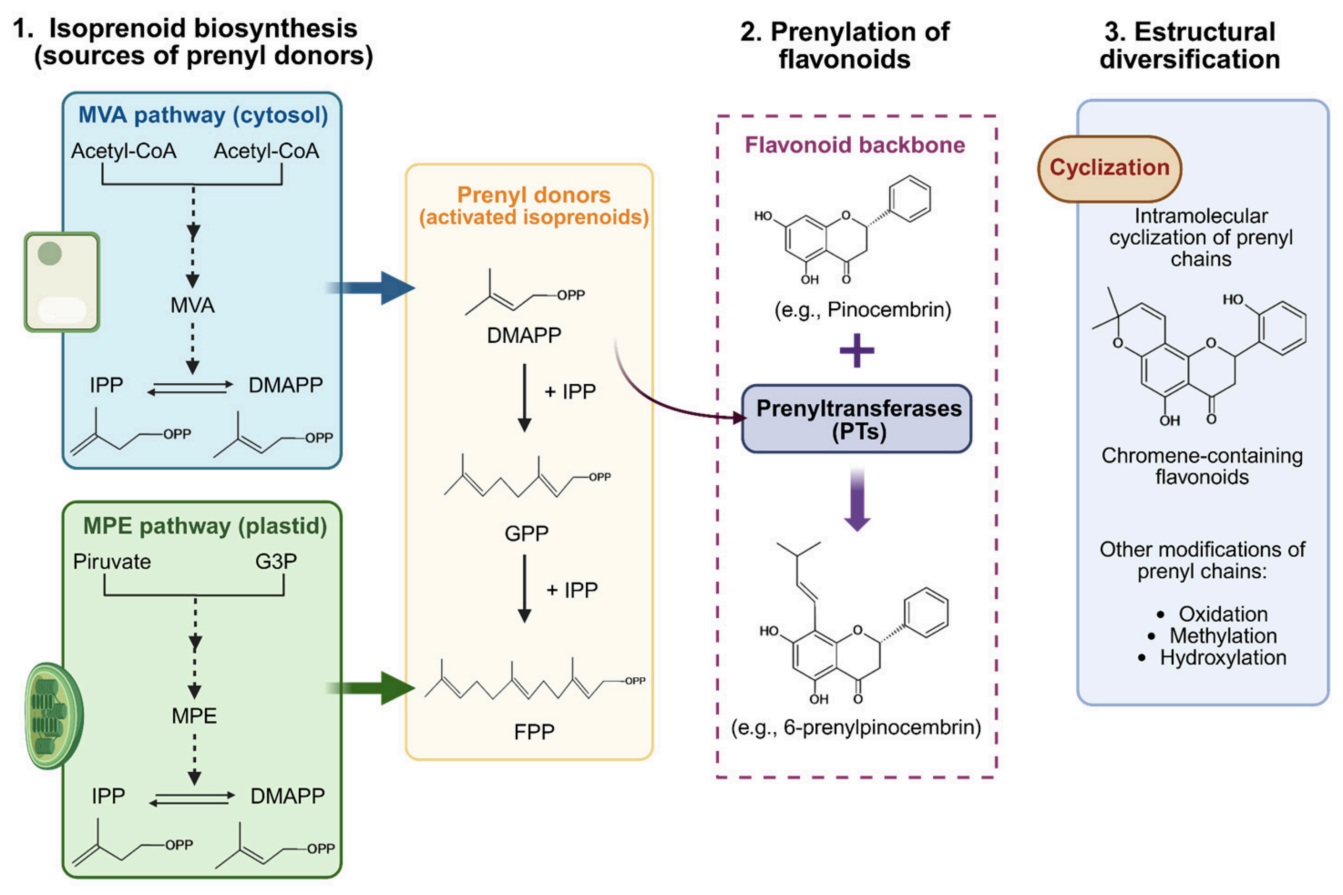
Simplified biosynthetic origin and structural diversification of prenylated flavonoids in the genus *Dalea*. The mevalonate (MVA) and methylerythritol phosphate (MEP) pathways generate the activated isoprenoid donors dimethylallyl diphosphate (DMAPP), geranyl diphosphate (GPP), and farnesyl diphosphate (FPP), which are transferred to flavonoid scaffolds by prenyltransferase enzymes (PTs). Prenylation constitutes a major diversification step in flavonoid biosynthesis and may be followed by additional structural modifications, including intramolecular cyclization of prenyl side chains to produce chromene-containing flavonoids. Created in BioRender. Biorender, B. (2026) https://BioRender.com/h6hh4nh (accessed on 3 June 2025).

The position and number of prenyl substituents significantly influence the physicochemical properties and biological activity of flavonoids. The prenyl moieties involved in flavonoid prenylation originate from the isoprenoid biosynthetic pathways, namely the mevalonate (MVA) and methylerythritol phosphate (MEP) pathways, which generate the activated isoprenoid donors DMAPP, geranyl diphosphate (GPP), and farnesyl diphosphate (FPP). These activated donors are transferred to flavonoid acceptors by membrane-bound prenyltransferases, representing one of the major mechanisms responsible for the structural diversification of plant specialized metabolites [57,58]. Following prenylation, flavonoids may undergo additional enzymatic tailoring reactions that further increase their structural diversity. These include oxidation, methylation, glycosylation, and intramolecular cyclization of prenyl or geranyl side chains, frequently resulting in the formation of fused pyran (chromene) or furan rings [59]. These post-prenylation modifications generate structurally complex metabolites that often exhibit biological activities distinct from those of their non-cyclized counterparts. Several chromene-containing flavonoids identified in *Dalea*, particularly from *D. boliviana*, exemplify this structural diversification and illustrate the remarkable chemical versatility of the genus [18]. The biosynthetic origin of prenyl donors and the principal diversification steps leading to prenylated flavonoids are summarized in Figure 5.

The prenylated flavonoids were classified into 6 categories: prenylated flavones, prenylated flavanones, prenylated chalcones, prenylated isoflavones, prenylated flavans and isoflavans, and prenylated flavonostilbenes and biflavonoids according to their structural features [51]. Prenylated flavonoids are generally more lipophilic than their non-prenylated counterparts due to the presence of hydrophobic prenyl or geranyl groups. This increased lipophilicity enhances their affinity for biological membranes and membrane-associated proteins [55]. In particular, prenylated flavonoids have been reported to interact with membrane transport proteins such as P-glycoprotein (P-gp), an ATP-dependent efflux transporter involved in multidrug resistance [60]. As a result, prenylated flavonoids can inhibit P-glycoprotein, potentially increasing the intracellular accumulation of therapeutic agents and enhancing the pharmacological efficacy of flavonoids and other bioactive compounds. Consequently, these metabolites have attracted considerable interest as lead structures for pharmacological research and drug discovery [61].

In addition to normal prenylation, reverse prenylation has also been reported in flavonoids. In reverse prenylated flavonoids, the isoprenyl unit is attached in an inverted orientation compared to normal prenylation. For example, prenylated flavanones isolated from *Dalea* species, particularly *Dalea elegans*, *Dalea boliviana*, and *Dalea pazensis*, are a rich source of bioactive compounds featuring unique structural modifications, including reverse prenylation, in which the dimethylallyl unit is attached through the tertiary carbon rather than the primary carbon. These compounds have demonstrated valuable pharmacological activities, including potent antifungal activity against *Candida albicans* and neuroprotective properties [62,63,64]. It should be noted that the prenylated flavanones originally reported from *Dalea elegans* as 6-prenylpinocembrin (6P) and 2′-4′-dihidroxy-5′-(1,1-dimethylallyl)-6-prenylpinocembrin) (6PP) [65] were later structurally reassigned as 8-prenylpinocembrin (8P) **(1)** and 2′-4′-dihidroxy-5′-(1,1-dimethylallyl)-8-prenylpinocembrin) (8PP) **(2)**, respectively, following detailed two-dimensional NMR analyses by Peralta et al. 2014 [16]. Therefore, the revised nomenclature is adopted throughout this review to maintain consistency with the current literature.

**Figure 6:**
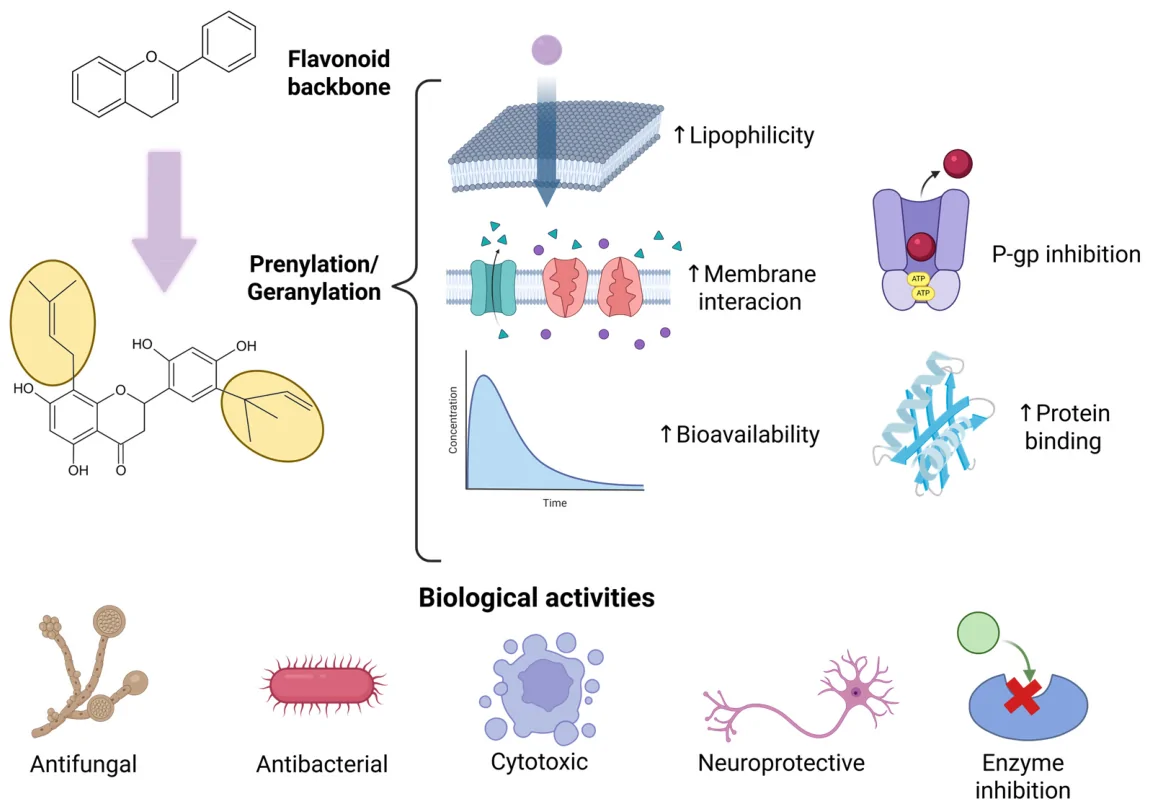
Relationship between prenylation and the biological activity of flavonoids. Prenylation and geranylation introduce hydrophobic isoprenoid groups into the flavonoid scaffold, increasing lipophilicity and enhancing interactions with biological membranes and membrane-associated proteins. These structural modifications improve membrane permeability, protein binding affinity, and modulation of molecular targets such as efflux pumps and enzymes. Consequently, prenylated flavonoids from the genus *Dalea* exhibit enhanced biological activities, including antifungal [66], antibacterial [67], cytotoxic [68], neuroprotective [64], and enzyme-inhibitory effects [63]. Created in BioRender. Biorender, B. (2026) https://BioRender.com/g1fpeva (accessed on 3 June 2025).

Prenylated flavonoids are also considered chemotaxonomic markers in several plant families, particularly Fabaceae and Moraceae. The presence of prenylated and geranylated flavonoids in the genus *Dalea* has been suggested as a characteristic chemical feature of the genus, which may be useful for chemotaxonomic classification and differentiation from other related genera within the Fabaceae family [17,53]. The relationship between prenylation and the biological activity of flavonoids is summarized in Figure 6.

##### Structure-Activity Relationships of Prenylated Flavonoids

Although prenylation is widely recognized for increasing the lipophilicity of flavonoids, accumulating evidence indicates that its contribution to biological activity extends far beyond improved membrane permeability [50]. The pharmacological profile and structure-activity relationships (SAR) of prenylated flavonoids are governed by a combination of structural variables, including the length and type of the isoprenoid chain, its position on the flavonoid scaffold, the mode of attachment (C- versus O-prenylation), and the occurrence of intramolecular cyclization, all of which contribute to the structural diversity of naturally occurring prenylflavonoids [59,69]. In addition, stereochemical configuration may further modulate biological activity in specific flavonoid subclasses, although comparative experimental evidence remains limited [70]. These structural features collectively influence the physicochemical properties, membrane partitioning, cellular uptake, metabolic stability, and interactions with biological targets, explaining why prenylated flavonoids frequently display greater potency than their non-prenylated analogs [71]. The prenyl moiety promotes stronger hydrophobic interactions with biological membranes and protein binding pockets, facilitating intracellular uptake and enhancing interactions with enzymes, receptors, and membrane-associated targets [55,72]. Consequently, prenylation has been associated with enhanced antimicrobial, antiparasitic, anti-inflammatory, cytotoxic, antioxidant, and enzyme-inhibitory activities across multiple flavonoid subclasses, highlighting its role as a key structural determinant of biological activity [52,55].

Among these variables, the structural characteristics of the isoprenoid substituent play a central role. The C5 prenyl (3,3-dimethylallyl) group is the most common prenyl substituent in naturally occurring flavonoids, whereas geranyl and farnesyl substituents further expand the structural and physicochemical diversity of these metabolites. Increasing prenyl chain length generally enhances lipophilicity and membrane affinity; however, excessive hydrophobicity may compromise aqueous solubility and reduce bioavailability. Therefore, biological activity depends not only on the presence of a prenyl moiety but also on the nature of the isoprenoid substituent, which influences membrane interactions and molecular recognition [52,54,55]. Increasing prenyl chain length does not necessarily translate into greater biological activity, as pharmacological performance depends on a balance between hydrophobicity, molecular flexibility, molecular shape, and target accessibility rather than maximal lipophilicity alone [73,74].

The position of prenylation represents another major determinant of biological activity. In flavonoids, prenyl groups are predominantly attached to the C-6 and C-8 positions of ring A, although substitution at C-3′ or C-5′ of ring B has also been reported [54]. The position of the prenyl moiety influences the three-dimensional architecture of the flavonoid scaffold, thereby affecting its physicochemical properties, interactions with biological membranes, and the spatial orientation of functional groups involved in molecular recognition [13,75]. In addition, C-prenylated flavonoids generally exhibit greater chemical and metabolic stability than O-prenylated derivatives because the carbon–carbon bond is considerably less susceptible to enzymatic cleavage. In contrast, O-prenylated flavonoids are more prone to oxidative deprenylation and other metabolic transformations, which may reduce their metabolic stability and biological persistence [76,77].

Another relevant structural modification is the intramolecular cyclization of the prenyl chain to generate fused pyran or furan rings, which alters the three-dimensional architecture of prenylated flavonoids. Rather than uniformly enhancing biological activity, cyclization exerts context-dependent effects that vary according to the flavonoid scaffold, the position of the prenyl substituent, and the biological target. For instance, cyclized derivatives have shown reduced antiplasmodial activity compared with their acyclic counterparts, whereas different substitution patterns have been associated with enhanced or diminished anti-inflammatory, cytotoxic, receptor-modulating, and enzyme-inhibitory activities. These findings highlight that the biological impact of prenyl cyclization is governed by the overall molecular architecture rather than by the cyclization process alone [51,78].

Finally, stereochemistry constitutes another potentially important, yet insufficiently explored, determinant of biological activity. Several flavanones isolated from *Dalea* occur as single naturally occurring stereoisomers, predominantly with the 2S configuration [18,79]. To our knowledge, comparative pharmacological studies evaluating the biological activities of R- and S-enantiomers of prenylated flavonoids from *Dalea* have not been reported. Nevertheless, considering that stereochemistry influences molecular recognition, enzyme selectivity, and receptor binding in many flavonoid subclasses, this aspect deserves greater attention in future phytochemical and medicinal chemistry investigations aimed at optimizing prenylated flavonoids as lead compounds for drug development [69].

Collectively, these findings emphasize that the pharmacological behavior of prenylated flavonoids arises from the combined effects of prenyl chain architecture, substitution pattern, and molecular conformation rather than from prenylation alone. Accordingly, the diversity of prenylated flavanones, chalcones, and isoflavones reported in *Dalea*, the genus represents an excellent model for future comparative SAR studies aimed at identifying the structural determinants underlying its diverse biological activities.

#### 3.1.3. Isoflavones and Isoflavans

Isoflavones and isoflavans are important subclasses of flavonoids that are widely distributed in the Fabaceae family. Unlike flavones and flavonols, in which the B ring is attached to position C-2 of the flavonoid skeleton, isoflavonoids are characterized by the migration of the B ring to position C-3, resulting in a different structural framework and biological properties [80]. This structural rearrangement is catalyzed by isoflavone synthase and represents a key step in isoflavonoid biosynthesis, which is particularly common in leguminous plants [81]. Isoflavonoids include several subclasses such as isoflavones, isoflavans, pterocarpans, and rotenoids, many of which have been reported in species of the genus *Dalea*. These compounds are mainly found in roots and underground tissues, although they may also occur in aerial parts [81]. Isoflavonoids are considered important defense compounds in legumes and often act as phytoalexins produced in response to microbial infection, environmental stress, or herbivory [82]. Isoflavones are one of the most common subclasses of isoflavonoids and typically include compounds such as formononetin, biochanin A, genistein, and daidzein derivatives [83]. These compounds have been reported in several species of the Fabaceae family and are known for their antioxidant, antimicrobial, anti-inflammatory, estrogenic, and anticancer activities. Isoflavones have attracted considerable attention due to their potential role as phytoestrogens and their possible applications in the prevention of hormone-dependent diseases, cardiovascular diseases, and certain types of cancer [84,85,86]. Isoflavans are a subclass of isoflavonoids, often described as reduced derivatives of isoflavones, characterized by a saturated heterocyclic C ring. Unlike isoflavones, which possess a 3-phenylchromen-4-one structure with a double bond between C2 and C3 and a carbonyl group at C4, isoflavans have a 3-phenylchroman skeleton, where the heterocyclic C ring is saturated and no carbonyl group is present at position 4 [87]. These compounds are less common than isoflavones but have been reported in several Fabaceae species, including species of the genus *Dalea*. Isoflavans have been associated with antimicrobial, cytotoxic, and antioxidant activity, and some prenylated isoflavans have shown particularly strong biological activity due to increased lipophilicity and membrane interaction [88,89,90]. Recent phytochemical investigations of *Dalea nana* further highlight the structural diversity and biological relevance of prenylated isoflavonoids within the genus. In this species, several prenylated isoflavans, including the newly described verdeans A–C **(8,9)**, together with related isoflavonoid derivatives, were isolated from root extracts [90]. These compounds exhibited notable antibacterial and antifungal activities against methicillin-resistant *Staphylococcus aureus* (MRSA) and *Cryptococcus neoformans*, supporting the role of prenylated isoflavans as important bioactive metabolites in *Dalea*.

#### 3.1.4. Rotenoids

Rotenoids are a class of isoflavonoid-derived compounds commonly found in plants belonging to the Fabaceae family. Structurally, rotenoids are advanced isoflavonoid derivatives characterized by a complex, fused, and generally cis-oriented tetracyclic core system (often denoted as A/B/C/D rings). They are formed in plants through the oxidative cyclization of isoflavonoid precursors, specifically 2′-methoxyisoflavones or 2′-hydroxyisoflavones, which introduces an additional heterocyclic ring (the B/C ring bridge) [91,92]. Rotenoids have been reported in several species of the genus *Dalea*, particularly in root and aerial extracts. For example, phytochemical investigations of *Dalea searlsiae* led to the isolation of known rotenoids along with isoflavones and other phenolic metabolites. These compounds were associated with antimicrobial and antiinsectan activities, suggesting that rotenoids may contribute to plant defense mechanisms against herbivores and microorganisms [30]. Similarly, studies on *Dalea ornata* identified rotenoids such as deguelin **(10)** and tephrosin **(11)**, in addition to flavonoid derivatives, further supporting the occurrence of rotenoid-type metabolites within the genus [40].

#### 3.1.5. Chalcones

Chalcones are open-chain flavonoids characterized by a C6-C3-C6 skeleton composed of two aromatic rings (A and B) connected by an α,β-unsaturated carbonyl system. Hydrogenation of the double bond yields saturated derivatives known as dihydrochalcones, in which the α,β-unsaturated ketone is converted into a saturated alkanone system. Chalcones are key biosynthetic intermediates in the flavonoid pathway and play a central role in plant secondary metabolism [93,94]. Although chalcones are best known as floral pigments, they are widely distributed throughout different plant tissues, including bark, heartwood, fruits, leaves, and roots. As specialized metabolites, they play important ecological and physiological roles, contributing to plant defense, adaptation, and regulatory processes. Their reported functions include attracting pollinators through pigmentation, protecting against microbial pathogens, filtering ultraviolet (UV) radiation, and deterring herbivorous insects [95]. Phytochemical investigations have identified several chalcones in species of the genus *Dalea*, including numerous prenylated derivatives that contribute substantially to the chemical diversity of the genus. For example, phytochemical investigations of *Dalea frutescens* led to the isolation of new prenylated chalcones such as sanjuanolide **(12)** and sanjoseolide **(13)**, which showed cytotoxic and antiproliferative activity against prostate cancer cell lines. Mechanistic studies suggested that these compounds affected cell cycle progression and mitotic spindle formation, indicating potential antitumor activity [68,96]. Chalcones isolated from *Dalea versicolor* have also been studied for their antibacterial activity. Some chalcones were found to enhance the activity of conventional antibiotics against bacterial strains, suggesting a potential role as antibiotic potentiators and resistance-modifying agents [97]. Overall, chalcones obtained from *Dalea* species have demonstrated a range of biological activities, particularly antitumor, antibacterial, and anti-inflammatory effects. These activities are frequently associated with prenylated chalcone structures, in which prenyl or geranyl substituents increase lipophilicity and enhance interactions with biological targets. More broadly, chalcones are considered privileged scaffolds in medicinal chemistry because their structural simplicity, synthetic versatility, and broad spectrum of biological activities have made them attractive templates for drug discovery. Although only a limited number of *Dalea*-derived chalcones have been pharmacologically characterized, the available evidence suggests that they represent valuable starting points for the development of new antimicrobial and anticancer agents, warranting further mechanistic and in vivo investigations [98].

#### 3.1.6. Stilbenes

Stilbenes are phenolic compounds characterized by a C6-C2-C6 structure consisting of two aromatic rings connected by an ethylene bridge. These compounds are produced through the phenylpropanoid pathway by the enzyme stilbene synthase and are structurally related to chalcones and flavonoids. Stilbenes are well known for their role as phytoalexins and defense compounds in plants [99]. Stilbenes have also been reported in several species of the genus *Dalea*, particularly in *Dalea purpurea*, where phytochemical investigations led to the isolation of geranyl-substituted stilbenes such as pawhuskins A **(14)**, B **(15)**, and C **(16)**. These compounds belong to a group of prenylated stilbenes that contribute to the chemical diversity of the genus and represent structurally unique natural products. Some of these stilbenes have shown biological activity, including affinity for opioid receptors, highlighting their pharmacological relevance and potential as lead compounds for drug discovery [39,100].

#### 3.1.7. Terpenoids and Essential Oils

**Figure 7:**
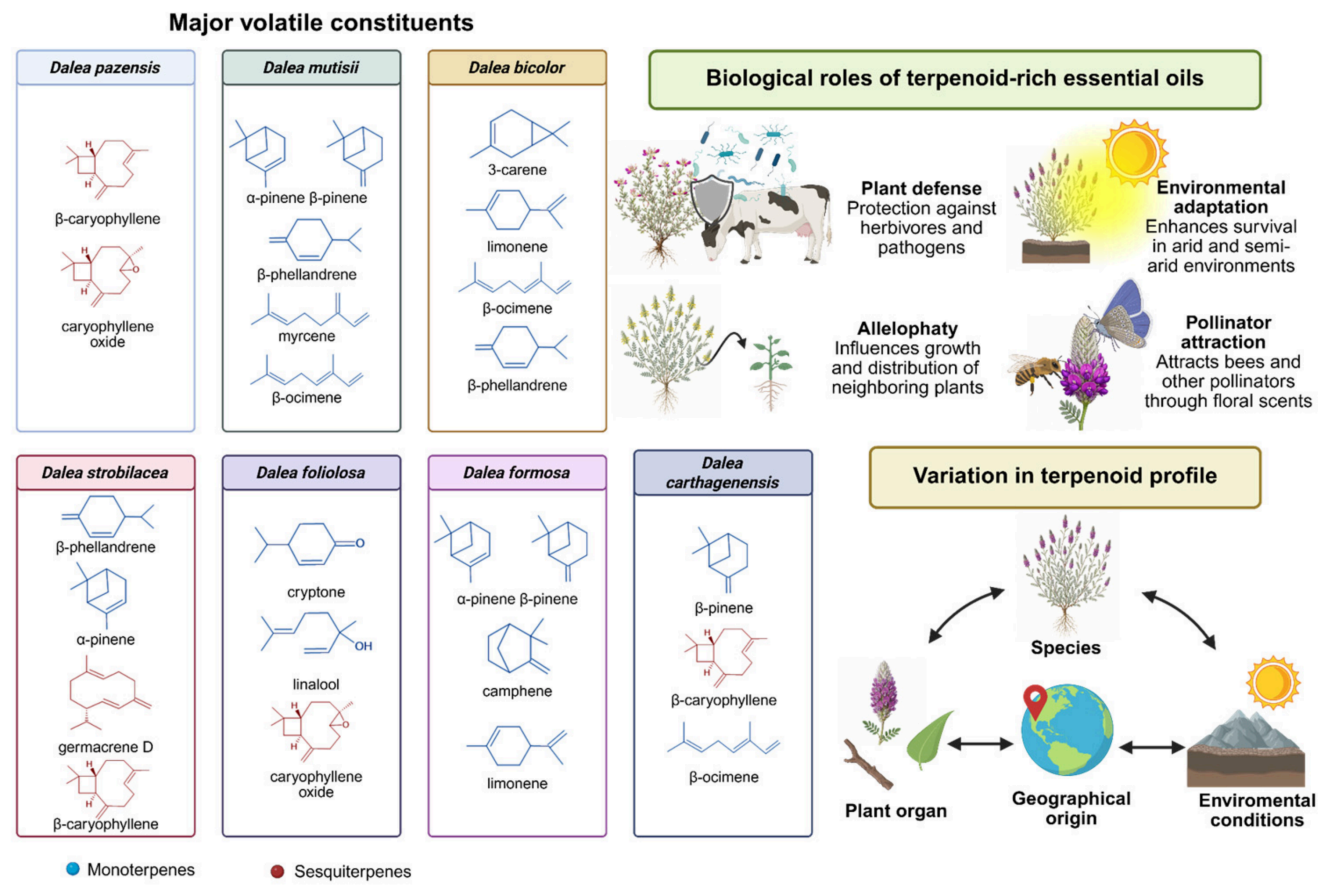
Major volatile terpenoid constituents and biological significance of essential oils reported in the genus *Dalea*. The composition of essential oils varies considerably among species, reflecting differences in plant organ, geographical origin, and environmental conditions. Most *Dalea* species produce essential oils dominated by monoterpenes or mixed mono- and sesquiterpene profiles. These volatile terpenoids contribute to ecological interactions, plant defense, environmental adaptation and pollinator attraction. Created in BioRender. Biorender, B. (2026) https://BioRender.com/c5pm3er (accessed on 3 June 2025).

Terpenoids represent one of the largest classes of plant secondary metabolites and are derived from isoprene units through the mevalonate (MVA) and methylerythritol phosphate (MEP) pathways. Terpenoids are commonly classified into monoterpenes, sesquiterpenes, diterpenes, and triterpenes depending on the number of isoprene units. These compounds play important ecological roles in plants, including defense against herbivores and pathogens, attraction of pollinators, and adaptation to environmental stress [101,102]. In the genus *Dalea*, terpenoids have mainly been reported as components of essential oils obtained from aerial parts, leaves, and flowers of several species. Essential oils from *Dalea* species are generally rich in monoterpenes and sesquiterpenes, which contribute to the aromatic properties of these plants and may be associated with their biological activities [15]. Essential oils from *Dalea* species are characterized predominantly by mixtures of mono- and sesquiterpenes, although the relative abundance of these constituents varies considerably among species. In most species examined to date, including *Dalea mutisii*, *Dalea bicolor*, *Dalea foliolosa*, *Dalea formosa*, *Dalea strobilacea* and *Dalea carthagenensis*, the volatile fraction is dominated by monoterpenes or contains a balanced mixture of mono- and sesquiterpenes. In contrast, the essential oil of *D. pazensis* is distinguished by an unusually high proportion of sesquiterpenes, with β-caryophyllene representing the major constituent (49.8%), followed by lower amounts of other sesquiterpenes. This compositional pattern highlights the remarkable chemical diversity of essential oils within the genus and suggests that *D. pazensis* represents a sesquiterpene-enriched profile compared with other *Dalea* species reported to date [43]. In *Dalea mutisii*, the essential oil is dominated by monoterpene hydrocarbons such as α-pinene, β-pinene, β-phellandrene, myrcene, and β-ocimene, which together represent the majority of the oil composition [15]. Similarly, the essential oil of *Dalea bicolor* contains monoterpenes such as 3-carene, limonene, ocimene, and phellandrene as major components [10]. In contrast, the essential oil of *Dalea strobilacea* contains both monoterpenes and sesquiterpenes, with β-phellandrene, α-pinene, germacrene D, and β-caryophyllene as major constituents [103]. Essential oil from *Dalea foliolosa* has been reported to contain oxygenated monoterpenes such as cryptone and linalool, as well as sesquiterpenes like caryophyllene oxide [104]. In another study, the chemical composition of the essential oil of *Dalea formosa* was determined, with α-pinene identified as the major constituent. High amounts of β-pinene, camphene, and limonene were also detected [42]. The volatile fractions and essential oils from leaves and branches of *Dalea carthagenensis* collected in northern Colombia were also chemically characterized. In this species, the essential oil was dominated by monoterpene hydrocarbons, with β-pinene, β-caryophyllene and (E)-β-ocimene identified as the major constituents. These findings further support that monoterpene-rich essential oils are common within the genus *Dalea*, although their composition may vary depending on the species, plant organ, and geographical origin [105]. The production of volatile terpenoids in *Dalea* species may also be related to their adaptation to arid and semi-arid environments, where volatile compounds participate in plant defense against herbivores, microbial pathogens, and environmental stress. In addition, essential oils may be involved in plant-plant interactions, pollinator attraction, and allelopathic effects [106,107]. Therefore, terpenoids and essential oils represent an important component of the secondary metabolism of the genus *Dalea* (Figure 7).

## 4. Traditional and Potential Health Applications

Species of the genus *Dalea* have been used in traditional medicine by Indigenous and rural communities across North, Central, and South America. Ethnobotanical reports indicate that several *Dalea* species have been traditionally used to treat gastrointestinal disorders, inflammation, infections, respiratory conditions, skin diseases, and wounds [9].

In Mexico, several *Dalea* species are used in traditional medicine. *Dalea carthagenensis* (Jacq.) J. F. Macbr., commonly known as “escobilla”, is used as an anti-inflammatory and to treat gastrointestinal infections. In Yucatán, Mexico, the leaves are applied as poultices to treat skin diseases and wounds caused by venomous animals [12,105]. *Dalea foliolosa* is traditionally boiled to prepare aqueous infusions used for the treatment of type 2 diabetes mellitus, and it is also applied locally to treat contusions and abrasions. This species is commonly found along roadsides and rainfed crop fields from Mexico to Honduras [9,104]. Another notable example is *Dalea bicolor*, locally known as “engordacabra”, which is widely used for gastrointestinal problems such as diarrhea, vomiting, and digestive disorders. In Aguascalientes, Mexico, it is traditionally administered as a tea prepared from branches, sometimes combined with other medicinal plants such as *Helianthemum glomeratum* and *Buddleja scordioides* and given to infants suffering from diarrhea or digestive problems [10,108,109].

Several *Dalea* species have also been used in Native American traditional medicine in the United States. *Dalea purpurea* Vent., commonly known as purple prairie clover, has been widely used by tribes such as the Pawnee, Dakota, and Plains Indians to treat heart disease, diarrhea, measles, pneumonia, and other ailments. The roots were typically pulverized, steeped in hot water, and administered as an infusion [14,39]. *Dalea compacta* (bush prairie clover) has been used by the Zuni people to treat stomachaches and to prepare poultices applied to sores and skin rashes [110]. Similarly, *Dalea formosa* (feather plume) has been utilized by several Native American groups, including the Jemez, Keres, Hopi, Pueblo, and Apache tribes. It has been used as a cathartic, emetic, analgesic, and for the treatment of influenza and other respiratory infections [41,97]. In addition, *Dalea aurea* (golden prairie clover) was used by the Dakota people in the form of infusions or decoctions to treat dysentery, stomachaches, and colic [89,111]. Another species, *Dalea polyadenia*, has been used by Native communities of the Colorado Desert not only for medicinal purposes but also for traditional practices such as dyeing fibers and coloring animal skins. Medicinally, hot teas prepared from fresh or dried stems have been used to treat colds, coughs, and infectious diseases such as smallpox [9]. Overall, Native American uses of *Dalea* species are mainly associated with gastrointestinal disorders, respiratory diseases, infections, skin conditions, and general inflammatory processes, suggesting the presence of bioactive compounds with antimicrobial, anti-inflammatory, and analgesic properties.

In South America, particularly in the Andean region of Chile and Peru, *Dalea strobilacea* Barneby is traditionally used to treat gastrointestinal smooth muscle spasms and digestive disorders such as stomach distress and indigestion. Its infusion is also consumed as a mild breakfast tea known as “hierba de chil” [103]. Another important species is *Dalea mutisii* Kunth, an Andean shrub distributed in Colombia, Ecuador, and Peru. In Ecuador, the flowers are used to treat pneumonia, while in Colombia the leaves and flowers are prepared as a decoction used as an insect repellent to eliminate lice and fleas [15].

Therefore, traditional medicinal uses of *Dalea* species are mainly associated with gastrointestinal disorders, infections, inflammation, respiratory diseases, skin conditions, and wound healing. These uses are consistent with the phytochemical composition of the genus, which includes flavonoids, isoflavonoids, chalcones, stilbenes, rotenoids, and terpenoids, many of which are known to possess antimicrobial, anti-inflammatory, antioxidant, and cytotoxic activities. Therefore, ethnopharmacological knowledge supports the pharmacological potential of the genus *Dalea* and highlights the importance of further phytochemical and pharmacological studies.

## 5. Biological and Pharmacological Activities of *Dalea* Genus

### 5.1. Antibacterial Effects

Several extracts and isolated compounds from species of the genus *Dalea* have demonstrated antibacterial activity in vitro. For example, three prenylated flavonoids isolated from *Dalea scandens* (Miller) R. Clausen var. *paucifolia* demonstrated activity *against Staphylococcus aureus*. The compounds 2(S)-5′-(1‴,1‴-dimethylallyl)-8-(3″,3″-dimethylallyl)-2′,4′,5,7-tetrahydroxyflavanone, 2(S)-5′-(1‴,1‴-dimethylallyl)-8-(3″,3″-dimethylallyl)-2′-methoxy-4′,5,7-trihydroxyflavanone, and 5′-(1‴,1‴-dimethylallyl)-8-(3″,3″-dimethylallyl)-2′,4′,5,7-tetrahydroxyflavone inhibited both susceptible and methicillin-resistant strains (ATCC 29213 and 43300), with minimum inhibitory concentrations (MICs) ranging from 1.56 to 3.13 µg/mL, compared to ciprofloxacin (MIC = 0.195 µg/mL). Structure-activity relationship analysis indicated that dihydroxylation on the B ring is critical for antimicrobial potency [67]. Similarly, compounds recovered from *Dalea versicolor* have been tested against *S. aureus* and *Bacillus cereus* individually or combined with known antimicrobials. The isolated molecules were a prenylated isoflavanone, two prenylated flavanones, a chalcone, a pterocarpin, and two stilbenes. Notably, the chalcone and stilbene enhanced the activity of berberine and conventional antibiotics, likely through inhibition of the NorA multidrug efflux pump, suggesting a potential role as resistance-modifying agents [97]. Although the results are consistent with efflux pump inhibition, the reduction in MIC values observed in the efflux pump mutant strain suggests that additional antibacterial mechanisms may also contribute to the observed activity. Therefore, the possibility that mechanisms other than efflux pump inhibition are involved cannot be excluded.

Condensed tannins from *Dalea purpurea* have also shown notable antibacterial activity against *Escherichia coli*, with MIC values reported to be four- to six-fold lower than those of other plant-derived tannins. Pharmacologically, these compounds act in a concentration-dependent manner, increasing bacterial lag phase and reducing growth rates within the range of 25–200 µg/mL, displaying predominantly bacteriostatic effects at concentrations below 400 µg/mL. Mechanistic studies indicate that *Dalea*-derived tannins possess a strong protein-precipitating capacity, leading to increased outer membrane permeability at low concentrations (10 µg/mL), and induction of bacterial cell aggregation at higher concentrations (50–200 µg/mL). Additionally, treatment at 50 µg/mL alters membrane lipid composition by decreasing unsaturated fatty acids, while ultrastructural analyses reveal disruption of the outer membrane and formation of electron-dense complexes on the cell surface. An important methodological limitation is that MIC assays were performed in M9 minimal medium rather than in the standardized MHB. Because the physiological state of *E. coli* is strongly influenced by nutrient availability [112], MIC values obtained in M9 may not be directly comparable with those reported using standardized susceptibility testing conditions [113,114]. In another research, the geranylated flavanone malheuran A **(3)**, produced by elicited hairy root cultures of *Dalea purpurea*, exhibits potent antibacterial activity primarily against Gram-positive pathogens. The compound demonstrated MIC values ranging from 1.56 to 6.25 µg/mL against multiple strains, including *Staphylococcus aureus*, *Enterococcus faecalis*, *Enterococcus faecium*, *Bacillus subtilis*, *Micrococcus luteus*, and *Staphylococcus epidermidis*. Notably, malheuran A **(3)** showed activity against multidrug-resistant bacteria, including methicillin-resistant *S. aureus* (MRSA) and vancomycin-resistant enterococci (VRE), with MIC values of 3.12 µg/mL. In contrast, no significant activity was observed against Gram-negative bacteria (MIC > 100 µg/mL). These findings support the potential of *Dalea purpurea* as a source of prenylated flavonoids with selective and potent antibacterial activity against clinically relevant Gram-positive pathogens, including resistant strains [14].

Phytochemicals from *Dalea searlsiae* roots exhibit potent antibacterial activity, particularly prenylated flavanones. Fractionation yielded malheurans A-D **(3)**, along with known flavanones and prostratol F, which demonstrated strong activity against *Streptococcus mutans*, *Bacillus cereus*, and both oxacillin-sensitive and -resistant *Staphylococcus aureus*. These compounds showed MIC values ranging from 2.0 to 5.3 µg/mL against *S. mutans*, 2.3–8.0 µg/mL against *B. cereus*, and 3.1–6.8 µg/mL against *S. aureus* strains, including resistant phenotypes. Comparable activity against sensitive and resistant strains suggests that the antibacterial activity is unlikely to depend on β-lactam susceptibility mechanisms. In contrast, aerial-derived metabolites were inactive, indicating localization of antibacterial activity in root tissues [30].

Recently studies on *Dalea nana* identified prenylated isoflavans such as verdeans A-C **(8,9)** and related metabolites. Several of the isolated metabolites showed activity against MRSA, with MIC values ranging from 8.9 to 25.0 µM. Notably, verdean A **(8)** showed enhanced potency, with an MIC of 12.9 µM, supporting the antibacterial potential of prenylated isoflavans from *D. nana* against resistant Gram-positive pathogens [90].

In another study, three previously undescribed prenylated flavanones were isolated from *Dalea albiflora*, albiflorans A-C, together with six known flavanones and pterocarpans. The antimicrobial evaluation demonstrated potent activity against clinically relevant Gram-positive pathogens, including methicillin-resistant *Staphylococcus aureus* (MRSA) and vancomycin-resistant *Enterococcus faecalis* (VRE). Albifloran A **(4)** exhibited the highest antibacterial potency, with MIC values of 1.9 and 2.3 μM against MRSA and VRE [115]. Interestingly, the antibacterial potency of albiflorans was comparable to that previously reported for prenylated flavanones isolated from *D. searlsiae* and *D. scandens*, further supporting the importance of prenylated flavanones as one of the principal antibacterial metabolite classes identified within the genus.

Phytochemical investigations continue to expand the chemical diversity reported for the genus. For example, a recent study on *Dalea lanata* var. terminalis described three previously undescribed phenolic metabolites, designated lanatans A-C, together with nine known compounds. In contrast to lanatan A **(5)**, lanatan B displayed approximately ten-fold lower antibacterial activity, whereas lanatan C was essentially inactive against MRSA and VRE, illustrating how relatively small structural modifications can markedly influence antibacterial potency within this class of flavonoids [116].

Collectively, the available evidence consistently identifies prenylated flavanones, isoflavans, chalcones, and condensed tannins as the principal antibacterial metabolites reported in species of the genus *Dalea*. Most active compounds exhibit preferential activity against Gram-positive bacteria, including multidrug-resistant strains such as MRSA and VRE, whereas activity against Gram-negative bacteria is generally limited. Although several mechanisms have been proposed, including membrane disruption, protein precipitation, and inhibition of multidrug efflux pumps, mechanistic evidence remains available for only a small number of compounds. Moreover, direct comparison among studies is complicated by differences in bacterial strains, susceptibility testing protocols, culture media, and reporting units. Finally, despite the low MIC values reported for several purified metabolites, virtually all antibacterial studies have been limited to in vitro susceptibility assays, and no animal infection models or pharmacokinetic studies have been conducted to establish therapeutic efficacy or safety. Consequently, the antibacterial potential of *Dalea*-derived metabolites should be regarded as preliminary until further mechanistic and in vivo investigations become available.

### 5.2. Antifungal Effects

Antifungal activity within the *Dalea* genus has been widely reported against *Candida albicans*, *Candida glabrata*, and related species. Extracts from *Dalea formosa* exhibit moderate antifungal activity, with MIC values of approximately 20 µM against *C. glabrata* whereas no inhibitory activity was observed against the reference strain of *C. albicans* (MIC > 80 µg/mL). Likewise, the extract failed to inhibit previously characterized azole-resistant *C. albicans* isolates overexpressing CDR1 and/or CDR2, indicating limited antifungal activity against *C. albicans* under the experimental conditions evaluated [117]. Isolated isoflavonoids from *D. formosa*, particularly sedonan A, displayed enhanced antifungal activity, with MIC values as low as 1.9 µg/mL in transporter-deficient strains and 7.6 µg/mL in wild-type strains, although activity decreased significantly in strains overexpressing efflux transporters. Importantly, combinations of sedonan A with *ent*-sandwicensin showed synergistic antifungal effects, suggesting that these compounds may act as resistance-modifying agents [41].

Prenylated flavonoid obtained from *Dalea elegans*, 2′,4′-dihydroxy-5′-(1‴,1‴-dimethylallyl)-6-prenylpinocembrin (6PP), exhibited antifungal activity against azole-resistant *Candida albicans* (MIC = 150 µM) and inhibited rhodamine 6G efflux (IC_50_ = 119 µM), indicating modulation of multidrug transporters. Notably, it significantly potentiated fluconazole activity, reducing its MIC by more than 400-fold in resistant strains, effectively reversing azole resistance [66]. Another derivative, 2′,4′-dihydroxy-5′-(1‴,1‴-dimethylallyl)-8-prenylpinocembrin (8PP) **(2)**, demonstrated strong activity against *C. albicans* biofilms (SMIC_80_ = 100 µM), affecting both sensitive and resistant strains. Its mechanism involves induction of oxidative stress, increasing reactive oxygen and nitrogen species and triggering antioxidant defenses [118]. Moreover, 8PP **(2)** showed additive interactions with fluconazole (FICI = 0.61), reducing its minimum fungicidal concentration four-fold and achieving near-complete fungicidal effects. Mechanistic studies revealed inhibition of efflux-associated ATPase activity and competitive interaction with ATP, supporting its role as an efflux pump inhibitor. Preliminary in vivo toxicity studies showed moderate toxicity, with LD_50_ values of 245–357 mg/kg for 8PP **(2)** in mice. Overall, these findings highlight *Dalea*-derived 8PP **(2)** as a promising antifungal adjuvant capable of enhancing fluconazole efficacy and may help counteract efflux-mediated azole resistance, although further validation in diverse clinical isolates and in vivo models is required [119]. Interestingly, 8PP **(2)** exhibits dual redox behavior: prooxidant effects at moderate concentrations (50–100 µM) and antioxidant effects at higher concentrations (200–1000 µM), accompanied by structural alterations in biofilm architecture. These findings highlight *Dalea elegans* metabolites as candidates for targeting biofilm-associated infections and overcoming antifungal resistance [120]. In recent years, the compound 8PP **(2)** exhibits antifungal activity against azole-resistant *Candida albicans* through modulation of energy metabolism and efflux-associated ATPase activity. At 125 µM, 8PP **(2)** inhibited CDR (*Candida* Drug Resistance)-associated ATPase activity by 58%, comparable to fluconazole (61%), while their combination achieved enhanced inhibition (70%). The IC_50_ of 8PP **(2)** for ATPase inhibition was 78.6 µM, and notably, co-administration with 125 µM 8PP reduced the IC_50_ of fluconazole by three-fold, indicating potentiation. Kinetic analysis suggested a competitive interaction between 8PP **(2)** and ATP at the enzymatic site. While no significant cytotoxicity was observed at 125 µM, higher concentrations resulted in reduced cell viability (IC_50_ = 355 µM for 8PP **(2)**). Thus, *Dalea elegans*-derived 8PP **(2)** inhibits ATPase activity and enhances fluconazole activity, suggesting interference with ATP-dependent efflux mechanisms that may contribute to overcoming azole resistance [121]. Collectively, these studies indicate that 8PP **(2)** exerts antifungal activity through multiple complementary mechanisms, including inhibition of ATP-dependent efflux systems, disruption of biofilm formation, and induction of oxidative stress. Nevertheless, these mechanisms have been investigated exclusively under in vitro conditions, and their relative contribution to antifungal efficacy in vivo remains unknown.

In addition, antifungal activity has also been reported for *Dalea pazensis*. Phytochemical analysis of this species revealed the presence of prenylated phenolic compounds with inhibitory effects against fungal pathogens, particularly clinical isolates of *Candida albicans*, including an isolate harboring multidrug resistance-associated genes. A chloroform extract obtained from the roots exhibited a MIC of 125 μg/mL. At a concentration of 250 μg/mL, the extract inhibited the growth of both an azole-sensitive strain and an azole-resistant clinical isolate of *C. albicans*. The major compound identified, (2S)-5,7,2′,4′-tetrahydroxy-5′-(1‴,1‴-dimethylallyl)-8-prenylflavanone, contributes substantially for this antifungal activity [43]. Interestingly, the same MIC values were observed for both clinical isolates, including the isolate overexpressing multidrug resistance-associated genes. This finding suggests that the antifungal activity of the prenylated phenolic compounds may not be substantially influenced by the resistance mechanisms present in the tested isolate. However, because the study evaluated only two clinical isolates, the generalizability of these findings to resistant *Candida albicans* populations remains uncertain, highlighting the need for additional studies involving a larger number of well-characterized clinical isolates with diverse antifungal resistance profiles.

Additionally, metabolites from *Dalea nana* demonstrated significant antifungal activity. Several compounds inhibited the growth of *Cryptococcus neoformans* with MIC values between 6.7 and 37.0 µM, while verdean A **(8)** also showed activity against *Candida albicans* (MIC = 10.0 µM). The antifungal activity of these prenylated isoflavans supports the antifungal potential of *D. nana* as a source of bioactive compounds against reference strains of clinically relevant fungal species [90].

Collectively, the available evidence identifies prenylated flavonoids and isoflavonoids as the principal antifungal metabolites reported in species of the genus *Dalea*. Most studies have focused on *Candida albicans*, including selected azole-resistant isolates, whereas comparatively little information is available for other clinically relevant fungal pathogens. Several complementary mechanisms have been proposed, including inhibition of ATP-dependent efflux pumps, interference with ATPase activity, biofilm disruption, and modulation of oxidative stress. However, mechanistic evidence remains available for only a limited number of purified compounds, predominantly 8PP **(2)**, and it is still unclear whether these mechanisms are broadly shared among other *Dalea*-derived metabolites. Furthermore, direct comparison among studies is limited by differences in fungal strains, resistance phenotypes, experimental endpoints, and susceptibility protocols. Importantly, all antifungal studies conducted to date have been restricted to in vitro assays, with only preliminary toxicity evaluations available for selected compounds. Consequently, the antifungal potential of *Dalea*-derived metabolites should be regarded as preliminary until validated in well-designed animal infection models and pharmacokinetic studies.

### 5.3. Antiparasitic Effects

Antiparasitic activity has been reported for several metabolites isolated from species of the genus *Dalea*; however, the available evidence remains limited and varies considerably according to the parasite species, experimental model, and metabolite class investigated. Two compounds isolated from *Dalea aurea*, the isoflavan 3S(+)-7-methoxymanuifolin K and manuifolin K, were evaluated for antiparasitic activity in both in vitro and in vivo models against the amoeba *Naegleria fowleri*. Both compounds inhibited the growth of *Naegleria fowleri* trophozoites in vitro at 30 µM, producing growth inhibition comparable to that of amphotericin B after prolonged incubation. However, in a mouse model of primary amebic meningoencephalitis, administration of manuifolin K at a dose of 25 mg/kg/day did not protect mice from infection compared to the positive control [89]. The discrepancy between the in vitro activity and the lack of protection observed in vivo suggests that factors such as pharmacokinetics, bioavailability, or limited tissue distribution may restrict therapeutic efficacy. Nevertheless, these aspects have not yet been investigated experimentally.

In contrast, metabolites from the aerial parts of *Dalea ornata* exhibited notable anthelmintic activity against the hookworm *Ancylostoma ceylanicum* in an ex vivo hamster model. Bioassay-guided fractionation of the crude extract yielded phenolic metabolites, among which the rotenoids deguelin **(10)** and tephrosin **(11)** showed the highest potency, inducing complete worm mortality by day 4 at low micromolar concentrations (6.3 and 6.0 µM, respectively). In contrast, the newly identified (2S)-8-(3-methylbut-2-en-1-yl)-6,7,4′-trihydroxyflavanone displayed weak activity, reducing worm survival by 17% at 7.3 µM after 5 days. Fractionation enhanced activity, as medium-polarity fractions demonstrated increased potency compared to the crude extract, showing an approximately two-fold improvement in efficacy. These results identify rotenoids from *D. ornata* as the most active antiparasitic metabolites reported in the genus to date [40]. However, activity was evaluated only in an ex vivo hookworm assay, and no mammalian efficacy or toxicity studies have yet confirmed their therapeutic potential.

Phytochemical investigation of *Dalea parryi* roots led to the isolation of several isoflavonoids, including the newly described parryans A-C, along with chalcones and pterocarpans, which were evaluated for anthelmintic activity against *Ancylostoma ceylanicum*. in an ex vivo assay. Overall, most isolated metabolites exhibited only limited anthelmintic activity, with the most active metabolite reducing worm survival by approximately 17% at 50 µg/mL after 5 days, whereas most compounds produced minimal effects under the experimental conditions. In contrast, several metabolites showed considerable cytotoxicity toward murine splenocytes, reducing cell viability by 47–93% at the same concentration, and some compounds also induced mitogenic responses. These findings indicate that, despite the structural diversity of metabolites isolated from *D. parryi*, their anthelmintic activity remains modest in the ex vivo model evaluated. Furthermore, the concomitant cytotoxic effects underscore the importance of assessing compound selectivity and safety profiles alongside antiparasitic efficacy in future studies [122].

Collectively, the available evidence indicates that antiparasitic activity within the genus *Dalea* is highly variable and appears to depend strongly on both the parasite model and the metabolite class evaluated. Among the compounds investigated to date, rotenoids have demonstrated the greatest anthelmintic potency, whereas isoflavonoids and flavonoids generally exhibit more modest or inconsistent activity. Importantly, the available studies employ markedly different experimental systems, including protozoan trophozoites, ex vivo helminth models, and limited in vivo assays, making direct comparison of efficacy across studies difficult. Furthermore, the discrepancy between the in vitro activity observed for *Naegleria fowleri* and the lack of efficacy in the corresponding mouse model, together with the cytotoxicity reported for several *D. parryi* metabolites, underscores the importance of evaluating pharmacokinetics, selectivity, and safety alongside antiparasitic activity. Consequently, the therapeutic potential of *Dalea*-derived antiparasitic compounds remains preliminary and requires validation in well-designed animal models.

### 5.4. Antiinsecticide Effects

Antiinsectan activity has been reported for metabolites isolated from *Dalea searlsiae*, with both root- and aerial-derived compounds contributing to growth inhibition and mortality of the fall armyworm (*Spodoptera frugiperda*). Methanolic extracts from both roots and aerial parts markedly reduced larval growth, and subsequent bioassay-guided fractionation identified ten phenolic metabolites responsible for this activity. Notably, the rotenoids tephrosin **(11)** and milletosin exhibited the highest activity, causing greater than 90% reduction in larval growth along with significant mortality. Among flavanones, malheuran D also showed strong activity, inducing approximately 90% growth inhibition and 37% mortality. Although malheuran D exhibited insecticidal activity comparable to that of the rotenoids in terms of growth inhibition, larval mortality was lower, suggesting differences in their modes of action or toxicity. Overall, these findings suggest a differential ecological allocation of specialized metabolites within *Dalea searlsiae*, with aerial tissues contributing more prominently to antiinsectan defense through growth inhibition and increased larval mortality [30]. However, the available evidence is restricted to a single herbivorous insect species evaluated under short-term laboratory conditions. Consequently, it remains unclear whether the observed activity extends to other economically important insect pests or reflects a broader ecological defense strategy. Moreover, the mechanisms responsible for larval growth inhibition, the selectivity toward beneficial insects and other non-target organisms, and the efficacy of these metabolites under field conditions have not yet been investigated. Therefore, the antiinsectan potential of *Dalea*-derived metabolites should be regarded as preliminary until validated in additional insect models and ecologically relevant bioassays.

### 5.5. Anticancer Activity

Limited evidence from *Dalea frutescens* indicates that selected isoprenylated chalcones possess cytotoxic and antiproliferative activity in prostate cancer cell models. Two new isoprenylated chalcones, sanjuanolide **(12)** and sanjoseolide **(13)**, were isolated and evaluated against the prostate cancer cell lines PC-3 and DU145. These compounds exhibited relevant antiproliferative activity, with IC_50_ values of 11.0 ± 4 and 35.0 µM in PC-3 cells, and 7.0 ± 3 and 25.5 µM in DU145 cells, these findings indicate moderate-to-low micromolar antiproliferative activity under the in vitro conditions evaluated. Mechanistic studies further demonstrated that sanjuanolide **(12)** induced G2/M cell-cycle arrest and promoted the formation of abnormal mitotic spindles, suggesting interference with mitotic progression as a likely mechanism of action. In tubulin polymerization assays, sanjuanolide **(12)** increased the initial rate of tubulin polymerization without altering the final amount of polymerized tubulin, indicating a specific effect on microtubule dynamics [68]. These findings are consistent with the established role of mitotic disruption as a therapeutic strategy in cancer chemotherapy.

The cytotoxic activity of *Dalea* metabolites appears to be strongly influenced by structural features such as prenylation and chalcone scaffolds. Prenyl substituents may enhance lipophilicity, membrane permeability, and interactions with intracellular protein targets, while the α,β-unsaturated carbonyl system of chalcones can confer electrophilic properties and has been associated with interactions with nucleophilic protein residues in other chalcone derivatives; however, its contribution to the cytotoxic effects of sanjuanolide **(12)** and sanjoseolide **(13)** has not been demonstrated experimentally. Similarly, no direct comparison with non-prenylated analogs was performed, preventing firm conclusions regarding the contribution of prenylation to their antiproliferative potency [123,124]. Subsequent synthesis of sanjoseolide **(13)** analogues showed that structural modification of the scaffold can improve antiproliferative potency; however, these synthetic derivatives should be distinguished from metabolites naturally occurring in *Dalea* [125].

Collectively, the available evidence for anticancer activity in the genus *Dalea* remains limited to two chalcones from a single species evaluated in two prostate cancer cell lines. Although sanjuanolide **(12)** produced measurable effects on cell-cycle progression and microtubule dynamics, the absence of comparisons with non-malignant cells prevents assessment of cancer-cell selectivity. Moreover, apoptosis, clonogenic survival, metastatic behavior, pharmacokinetics, and antitumor efficacy in animal models have not been sufficiently characterized for these *Dalea*-derived compounds. Therefore, sanjuanolide **(12)** and sanjoseolide **(13)** should currently be regarded as preliminary cytotoxic lead structures rather than validated anticancer candidates. Additional studies using broader panels of malignant and non-malignant cells, molecular target validation, and well-designed in vivo tumor models are required to determine their pharmacological relevance.

### 5.6. Anti-Inflammatory Effects

Anti-inflammatory activity has been reported for a limited number of *Dalea*-derived metabolites, particularly prenylated flavonoids and chalcones, in cell-based models of LPS-induced inflammation. A recent study reported the anti-inflammatory potential of malheuran A **(3)**, a geranylated flavonoid produced in elicited hairy root cultures of *Dalea purpurea*. The anti-inflammatory activity was evaluated in LPS-stimulated RAW 264.7 macrophages by measuring nitrite production using the Griess assay. Malheuran A **(3)** significantly reduced nitrite accumulation in LPS-stimulated RAW 264.7 macrophages, with nitrite production decreasing to 31.11% of the LPS-treated control at 15 µM. Cell viability was assessed in parallel, supporting that the reduction in nitrite levels was not attributable to overt cytotoxicity under the conditions tested. Although the inclusion of a viability assay strengthens the interpretation of these findings, the anti-inflammatory evidence for malheuran A **(3)** is currently limited to a single cell line and a single inflammatory readout [14]. Additional studies examining cytokine production, signaling pathways, and in vivo inflammatory models are required to determine whether this effect extends beyond suppression of nitrite production. Additional evidence of anti-inflammatory activity within the genus *Dalea* has been reported for sanjuanolide **(12)**, a prenylated chalcone originally isolated from *Dalea frutescens*. In this study, the anti-inflammatory effects of (R)-, (S)-, and racemic sanjuanolide **(12)** were evaluated in lipopolysaccharide (LPS)-stimulated macrophages. Notably, the (R)-enantiomer exhibited potent inhibitory activity, significantly reducing the production of pro-inflammatory cytokines tumor necrosis factor-α (TNF-α) and interleukin-6 (IL-6), with an IC_50_ value of 1.2 and 1.1 µM, respectively. In contrast, the (S)-enantiomer showed markedly lower activity, highlighting the importance of stereochemistry in modulating biological effects. Furthermore, (R)-sanjuanolide **(12)** was shown to suppress the mRNA expression of inflammatory cytokines (TNF-α, IL-6, IL-1β), ICAM-1 (intercellular adhesion molecule-1), MACP-1 (mitochondrial anion carrier proteins-1), and COX-2 (cyclooxygenase-2) following LPS stimulation, consistent with modulation at the transcriptional level [96]. Collectively, the available evidence for anti-inflammatory activity in the genus *Dalea* remains limited but suggests that structurally distinct phenolic metabolites can modulate inflammatory responses in LPS-stimulated macrophages. The two studies differ substantially in the depth of mechanistic evaluation: malheuran A **(3)** has been assessed primarily through nitrite production, whereas sanjuanolide **(12)** has been examined using cytokine secretion, gene-expression endpoints, and stereochemical comparisons. Notably, the marked difference between the (R)- and (S)-enantiomers of sanjuanolide **(12)** provides direct evidence that stereochemistry influences its anti-inflammatory activity. However, both studies remain confined to in vitro macrophage models, and no animal models of inflammation have yet validated these effects. Therefore, the anti-inflammatory potential of *Dalea*-derived metabolites should be considered preliminary pending confirmation of molecular targets, pharmacokinetic behavior, safety, and in vivo efficacy.

### 5.7. Neuroprotective Effects

Neuroprotective activity has been investigated for a limited group of prenylated flavanones isolated from *Dalea elegans* and *Dalea pazensis*, primarily using in vitro models of oxidative and Alzheimer’s disease-related neurotoxicity. A study evaluating five prenylated flavanones isolated from these species demonstrated neuroprotective effects in two in vitro models of neurodegeneration, including primary cerebellar granule neurons and nerve growth factor (NGF)-differentiated PC12 cells exposed to oxidative stress. Among the evaluated compounds, flavanones (−)−(2S)-5,7,2′,4′-tetrahydroxy-5′-(1‴,1‴-dimethylallyl)-8-prenylpinocembrin **(2)** and (−)−(2S)-8-prenylpinocembrin **(1)** showed the highest protective activity, effectively preventing neuronal cell death induced by oxidative damage. SAR analysis revealed that specific structural features are critical for neuroprotective activity. In particular, the presence of an 8-prenyl group on ring A, together with either an unsubstituted B-ring or a 2′,4′-dihydroxy-5′-dimethylallyl substitution pattern, significantly enhanced biological activity. These findings highlight the importance of prenylation in modulating the neuroprotective potential of flavanones. The SAR analysis indicated that neuroprotective activity depended not simply on prenylation itself, but on the position of the prenyl substituent and the substitution pattern of the B ring. Among these, acetylcholinesterase (AChE) was experimentally validated, and the most active compounds exhibited inhibitory activity against this enzyme. Notably, glabranin showed inhibitory potency in the nanomolar range (IC_50_ 64 nM), surpassing the reference inhibitor physostigmine, suggesting a potential as a multi-target-directed ligand for neurodegenerative disorders [126].

More recently, the neuroprotective potential of prenylated flavanones from *Dalea* species has been further validated using a more complex in vitro Alzheimer’s disease model using neurotoxic astrocyte-conditioned media derived from 3xTg-AD mice. In this system, neuronal cultures exposed to the pathological microenvironment exhibited significant neurodegeneration, mimicking key features of Alzheimer’s disease. Of these metabolites, (-)-(2S)-5,7,2′,4′-tetrahydroxy-5′-(1‴,1‴-dimethylallyl)-8-prenylflavanone (8PP) **(2)**, showed the most potent neuroprotective effect, restoring neuronal survival at nanomolar concentrations (EC = 10 nM). Mechanistic studies demonstrated that this compound significantly reduced tau hyperphosphorylation, a hallmark of Alzheimer’s disease pathology, through inhibition of glycogen synthase kinase-3β (GSK-3β), a key enzyme involved in tau regulation. In vitro assays confirmed a concentration-dependent inhibition of GSK-3β activity, with an IC_50_ of approximately 43 ± 15 µM. Additionally, molecular docking and dynamic simulations revealed that 8PP **(2)** interacts with the ATP-binding site of GSK-3β, establishing hydrogen bonding interactions with key residues such as Asp133, Val135, and Asp200, providing a plausible binding mode consistent with the experimentally observed enzyme inhibition. Interestingly, beyond its direct neuronal effects, 8PP **(2)** also modulated astrocyte-mediated neurotoxicity. Notably, the concentration range associated with neuroprotection was substantially lower than that required for direct GSK-3β inhibition. This discrepancy suggests that inhibition of GSK-3β alone may not fully account for the cellular neuroprotective effect and that additional direct or indirect mechanisms may be involved. Direct treatment of 3xTg-AD-derived neurotoxic astrocytes with 8PP **(2)** reduced the subsequent neurotoxicity of their conditioned medium; however, 8PP **(2)** also exerted toxicity toward the astrocytes themselves. Therefore, the reduced neuronal toxicity cannot be interpreted exclusively as beneficial modulation of astrocyte function and may partly reflect a reduction in the viability or activity of the neurotoxic astrocyte population. Structure-activity relationship analysis indicated that the presence of a 5′-dimethylallyl and 4′-hydroxy substitution pattern on the B ring is essential for neuroprotective activity, while a 2′-hydroxy substituent at the B ring improved the activity [64].

Beyond direct neuroprotective effects, *Dalea* also produces metabolites with other CNS-related pharmacological activities. Pawhuskin A **(14)**, a prenylated stilbene isolated from *D. purpurea*, displayed opioid receptor affinity in vitro and was subsequently characterized as a non-nitrogenous opioid receptor antagonist with preferential binding toward the κ-opioid receptor [127].

Collectively, the available evidence indicates that selected prenylated flavanones from *D. elegans* and *D. pazensis* exert neuroprotective effects in several in vitro models. Although AChE inhibition and modulation of tau/GSK-3β signaling have been demonstrated, the marked difference between the concentrations required for cellular neuroprotection and direct GSK-3β inhibition suggests that additional mechanisms may contribute to these effects. Moreover, the reported toxicity of 8PP **(2)** toward reactive astrocytes complicates interpretation of its glial effects. All efficacy studies remain restricted to in vitro systems, and no evidence is currently available regarding brain exposure, blood–brain barrier penetration, pharmacokinetics, cognitive outcomes, or efficacy in animal models of neurodegenerative disease. Therefore, these prenylated flavanones should presently be regarded as neuroactive lead structures requiring further mechanistic and in vivo validation rather than established neuroprotective candidates.

### 5.8. Tyrosinase-Inhibitory and Antimelanogenic Activities

Tyrosinase is a copper-containing enzyme that plays a central role in melanogenesis by catalyzing the hydroxylation of L-tyrosine to L-DOPA and the subsequent oxidation of L-DOPA to dopaquinone. Dysregulated melanin production contributes to hyperpigmentation disorders such as melasma, freckles, and age-related hyperpigmentation. Accordingly, tyrosinase inhibition is widely investigated as a strategy for controlling excessive melanogenesis in dermatological and cosmetic applications [128]. Therefore, tyrosinase inhibitors negatively regulate melanin formation and have become increasingly important in medicinal, dermatological, and cosmetic applications aimed at treating hyperpigmentation and promoting skin tone uniformity.

Species of the genus *Dalea* have emerged as a promising source of natural tyrosinase inhibitors, particularly due to their high content of prenylated flavonoids. Phytochemical investigation of *Dalea elegans* led to the isolation of several flavonoids evaluated for in chemico anti-tyrosinase activity. Among the compounds evaluated, 8PP **(2)** was the most potent inhibitor, with an IC_50_ of 2.32 ± 0.01 μM, compared with 4.93 ± 0.01 μM for kojic acid under the same assay conditions. Kinetic studies demonstrated that 8PP **(2)** acts as a competitive inhibitor of tyrosinase, suggesting direct interaction with the catalytic site of the enzyme. Other flavonoids such as triangularin also exhibited relevant inhibitory activity with an IC_50_ value of 33.3 µM, in this case, showing a non-competitive inhibition of tyrosinase [16]. One of the most significant reports of tyrosinase inhibition within the genus *Dalea* involved the isolation of an isoprenoid-substituted flavanone from *Dalea elegans*, identified as dalenin (5,2′,4′-trihydroxy-2″,2″-dimethylchromene-(6,7:5″,6″)-flavanone). Dalenin showed strong inhibition of mushroom tyrosinase, with IC_50_ values of 0.26 and 18.61 μM using L-tyrosine and L-DOPA as substrates, respectively. Under the assay conditions employed, its monophenolase-inhibitory activity was reported to be 52-fold and 495-fold greater than that of hydroquinone and kojic acid, respectively. Kinetic analyses indicated that the compound acts as a reversible inhibitor, showing mixed-type inhibition with L-tyrosine and non-competitive inhibition with L-DOPA. Molecular modeling suggested that the 2′,4′-dihydroxy substitution pattern contributes to enzyme binding [129]. Recent studies further support the anti-tyrosinase potential of *Dalea elegans* at the extract level. Sequential extraction of roots using solvents of increasing polarity revealed that ethyl acetate extracts exhibited in vitro the highest tyrosinase-inhibitory activity among evaluated *Dalea* species, with an IC_50_ value of 1.44 µg/mL. This activity was attributed to the presence of prenylated flavanones, particularly 8PP **(2)**, which was identified as a key contributor to the observed inhibition. These findings highlight that not only isolated compounds but also enriched extracts of *D. elegans* possess strong tyrosinase-inhibiting activity, supporting the proposed role and relevance of prenylated flavonoids as major bioactive constituents in the genus [130].

Additional evidence of anti-tyrosinase activity in chemico within the genus was provided by *Dalea boliviana*, whose root *n*-hexane extract yielded three new prenylated flavanones together with the known compound obovatin. The compound (-)-(2S)-5,7,2′-trihydroxy-5′-(1‴,1‴-dimethylallyl)-8-prenylflavanone exhibited the highest potency, with an IC_50_ value of 3.79 µM, indicating notable inhibitory potency in the mushroom tyrosinase assay. Comparative analysis suggested that the substitution pattern, including prenylation and free phenolic hydroxyl groups, influences inhibitory activity [63].

Further support for the anti-tyrosinase potential of the genus was provided by phytochemical studies on *Dalea pazensis*, which led to the isolation of two new prenylated flavanones, pazentin A and pazentin B, together with previously known flavanones. Although the newly identified pazentins showed limited inhibitory activity, the major anti-tyrosinase effects were attributed to compounds 4′-hydroxy-2′-3methoxy-5′-(1‴,1‴-dimethylallyl)-8-prenylpinocembrin and 2′,4′-dihydroxy-5′-(1‴,1‴-dimethylallyl)-8-prenylpinocembrin **(2)**, which exhibited stronger enzyme inhibition. In addition, evaluation in cellular models demonstrated that active compounds were capable of reducing melanin production on murine B16 melanoma cells, providing cellular evidence of antimelanogenic activity for selected compounds [131]. Interestingly, reduction in cellular melanin content did not consistently parallel inhibition of intracellular tyrosinase, indicating that the antimelanogenic effects of some flavanones may involve additional mechanisms beyond direct tyrosinase inhibition [132].

Collectively, the available evidence identifies prenylated flavanones from *D. elegans*, *D. boliviana*, and *D. pazensis* as potent inhibitors of mushroom tyrosinase, with selected compounds also reducing melanogenesis in B16 cells. However, direct comparison among studies is complicated by differences in substrates, assay conditions, and biological endpoints. Moreover, potency against mushroom tyrosinase does not necessarily predict inhibition of mammalian intracellular tyrosinase, as illustrated by the *D. pazensis* studies. Because most evidence remains limited to enzyme-based assays and a single murine melanoma cell model, further studies using human melanocytes, reconstructed skin models, dermal permeation and safety assessments, and appropriate in vivo models are required before dermatological applications can be established.

### 5.9. Xanthine-Oxidase-Inhibitory Activity

Xanthine oxidase (XO)-inhibitory activity has been investigated for prenylated flavanones isolated from several *Dalea* species. Because XO catalyzes the formation of uric acid, its inhibition represents an established pharmacological strategy for the management of hyperuricemia and gout [133]. Among five prenylated flavanones isolated from *D. elegans*, *D. boliviana*, and *D. pazensis*, 8PP **(2)** was the most active, with an IC_50_ of 0.26 ± 0.07 µM, showing activity comparable to that of allopurinol under the assay conditions used. Other prenylated flavanones showed moderate to weak activity, highlighting the importance of specific structural features for enzyme inhibition. SAR analysis suggested that the 5,7,2′,4′-tetrahydroxy substitution pattern, an 8-prenyl group on ring A, and a 5′-dimethylallyl substituent on ring B favor XO-inhibitory activity. In contrast, compounds lacking this substitution pattern or presenting prenylation at alternative positions, such as C-6, exhibited significantly reduced or negligible activity. Molecular docking studies further supported the experimental results, showing that the most active compound established strong interactions within the active site of XO, including hydrogen bonding with key catalytic residues such as Glu802 and π–π interactions with Phe914. These interactions are essential for stabilizing the ligand within the enzyme’s active site and for inhibiting substrate oxidation [17].

Further advances in the identification of xanthine oxidase (XO) inhibitors from the genus *Dalea* were reported with the discovery of chromene flavanones from *Dalea boliviana*. In this in vitro study, three flavanone derivatives bearing a chromene moiety were isolated and evaluated for their XO-inhibitory activity. Notably, (2S)-5,2′-dihydroxy-6″,6″-dimethylchromeno-(7,8:2″,3″)-flavanone and (2S)-5,2′-dihydroxy-6″,6″-dimethylchromeno-(7,8:2″,3″)-3′-prenylflavanone **(7)** exhibited exceptionally high potency, with IC_50_ values in the nanomolar range (0.5 ± 0.01 nM and 1.7 ± 0.46 nM, respectively), significantly surpassing the reference inhibitor allopurinol. These results highlight chromene flavanones as one of the most potent classes of natural XO inhibitors reported within the genus *Dalea*. SAR analysis indicated that the presence of a chromene moiety in the A-ring, combined with hydroxyl and prenyl groups in the B-ring, played a crucial role in enhancing inhibitory activity. In particular, the rigidification of the molecular structure due to chromene formation appears to favor optimal positioning within the active site of XO. Docking predicted interactions with residues within the catalytic pocket, including hydrogen bonding and π–π contacts, providing a plausible binding mode consistent with the experimentally observed inhibition [18]. Collectively, the available evidence identifies prenylated and chromene-containing flavanones from *Dalea* as potent XO inhibitors in cell-free enzymatic assays. The large differences in activity among closely related analogs also indicate that inhibitory potency depends on a specific combination of hydroxylation, prenylation, and cyclization patterns rather than on prenylation or chromene formation alone. However, the evidence is currently limited to in vitro enzyme assays supported by molecular docking, and no cellular urate-production systems or animal models of hyperuricemia have been evaluated. Further studies addressing selectivity, cellular activity, pharmacokinetics, toxicity, and in vivo urate-lowering efficacy are required.

A summary of the principal bioactive metabolites isolated from *Dalea* species, together with their reported pharmacological activities and biological targets, is presented in Table 2.

## 6. Drug-likeness and Pharmacokinetic Considerations of *Dalea* Phytochemicals

Despite the remarkable pharmacological activities reported for numerous *Dalea* metabolites, information regarding their pharmacokinetic behavior remains scarce. To date, dedicated ADME studies are available for only a limited number of compounds isolated from the genus. Consequently, current knowledge relies largely on experimental or predictive pharmacokinetic analyses of structurally related flavonoids, rotenoids, chalcones, isoflavones, and prenylated stilbenes. Although these findings cannot be directly extrapolated to *Dalea* metabolites, they provide a useful framework for understanding the drug-likeness and pharmacokinetic behavior expected for chemically related compounds.

Flavanones structurally related to those identified in *Dalea* generally exhibit favorable drug-like characteristics. ADMET analyses of pinocembrin predict high intestinal absorption, blood–brain barrier permeability, and compliance with Lipinski’s rule of five, although potential hERG inhibition and CYP-mediated interactions warrant further toxicological evaluation [135]. Likewise, ADME predictions for the structurally related flavanone 2′,3,5,7-tetrahydroxyflavanone indicate low molecular weight, high gastrointestinal absorption, balanced lipophilicity, favorable aqueous solubility, and no Lipinski violations, suggesting good oral bioavailability and synthetic accessibility [136].

Similarly, the prenylated isoflavone 8-prenylgenistein (8PG), which is structurally related to the prenylated isoflavones identified in *Dalea*, has demonstrated a favorable predicted pharmacokinetic profile. In silico ADMET analysis revealed high human intestinal absorption (90.77%), extensive plasma protein binding (98.93%), and compliance with drug-likeness criteria, while also predicting a non-carcinogenic profile. Interestingly, 8PG was identified as a potential P-glycoprotein (P-gp) inhibitor, suggesting that, besides exhibiting favorable oral absorption, it may modulate drug transport and influence the pharmacokinetics of co-administered compounds [137].

Among structurally related metabolites, deguelin **(10)** represents one of the few compounds for which experimental pharmacokinetic data have been reported. In rats, deguelin **(10)** exhibited a relatively long plasma half-life (9.26 h), prolonged mean residence time (6.98 h), and extensive tissue distribution following both intravenous and intragastric administration, indicating sustained systemic exposure. The compound accumulated preferentially in highly perfused tissues and adipose tissue and was eliminated predominantly as metabolites through the feces, whereas only small amounts of unchanged compound were recovered in urine and feces. This suggests that deguelin undergoes extensive biotransformation while maintaining favorable tissue distribution characteristics, highlighting the importance of metabolism in determining the pharmacokinetic profile of rotenoid derivatives and supporting their potential as bioactive lead compounds despite their structural complexity [138].

Prenylated chalcones, another important class of specialized metabolites reported in *Dalea*, also exhibit promising predicted pharmacokinetic properties. ADME analysis of structurally related prenylated chalcones indicates favorable drug-like characteristics, including complete compliance with Lipinski’s rule of five, high predicted gastrointestinal absorption, and a bioavailability score of 0.55, supporting their suitability as orally administered small molecules [139]. Their moderate-to-high lipophilicity is expected to promote efficient membrane permeability while maintaining an adequate physicochemical balance for systemic absorption. Interestingly, the lack of predicted blood–brain barrier permeability suggests that these compounds may preferentially target peripheral tissues, potentially reducing the risk of central nervous system adverse effects. Although these predictions were generated for structurally related prenylated chalcones rather than compounds directly isolated from *Dalea*, they suggest that chalcone derivatives occurring in the genus may also possess favorable oral pharmacokinetic properties, a hypothesis that remains to be confirmed experimentally.

Because pharmacokinetic information on *Dalea* stilbenes is currently unavailable, predictive ADME studies of structurally related prenylated stilbenes from *Cannabis sativa* provide valuable indirect evidence. These compounds generally exhibit favorable oral absorption, moderate systemic exposure, and acceptable drug-like properties, while also demonstrating that hydroxylation, methoxylation, hydrogenation, and prenylation markedly influence pharmacokinetic performance. This variability indicates that even closely related prenylated stilbenes may differ substantially in their ADME behavior, emphasizing that dedicated pharmacokinetic studies are necessary before extrapolating these properties to pawhuskins A-C **(14,15,16)** [140].

Collectively, these findings suggest that several classes of phytochemicals structurally related to *Dalea* metabolites possess favorable drug-like and pharmacokinetic characteristics, including high predicted intestinal absorption, compliance with Lipinski’s rule of five, and acceptable oral bioavailability. Nevertheless, these observations are derived largely from structurally related compounds rather than metabolites directly isolated from *Dalea*. Consequently, dedicated pharmacokinetic, metabolic, and bioavailability studies are still required to validate the translational potential of *Dalea*-derived bioactive compounds.

## 7. Toxicological Considerations and Safety Profile

Although species of the genus *Dalea* have attracted considerable attention because of their diverse pharmacological activities, information regarding their toxicological profile remains limited and fragmented. This represents an important limitation for the potential therapeutic development of *Dalea*-derived metabolites, particularly considering that many of the bioactive compounds identified in the genus, such as prenylated flavonoids, chalcones, and isoflavonoids, possess high lipophilicity and strong interactions with biological membranes. These structural characteristics may contribute not only to their pharmacological properties but also to potential cytotoxic and toxicological effects [54,61].

Plants have historically been used in traditional medicine throughout the American continent, especially since pre-Hispanic times, for the prevention and treatment of numerous diseases [141]. However, despite the widespread ethnomedicinal use of several *Dalea* species, their complete biological and toxicological profiles remain poorly characterized. Current evidence suggests that the safety of flavonoids and related metabolites may vary considerably depending on factors such as dosage, exposure time, route of administration, and individual susceptibility. Although flavonoids are generally considered safe, previous studies have associated certain flavonoid derivatives with hepatotoxicity, nephrotoxicity, endocrine disruption, oxidative stress, mitochondrial dysfunction, and dose-dependent cytotoxicity [142,143]. Therefore, further toxicological studies focused on the specific metabolites present in *Dalea* species are necessary to better establish their safety profile and possible implications for human health.

One of the most extensively studied toxicological examples within the genus is the prenylated flavonoid 8PP **(2)** (originally reported as 6PP before structural reassignment), isolated from *Dalea elegans*. This compound was evaluated in vitro in rat liver mitochondria, where micromolar concentrations significantly altered mitochondrial respiration by affecting oxygen uptake during both state 3 and state 4 respiration. In addition, 8PP **(2)** induced a concentration-dependent decrease in mitochondrial membrane potential and inhibited key mitochondrial enzymes, including succinate dehydrogenase (SDH), NADH oxidase, and F0F1-ATPase. The available evidence indicates that 8PP **(2)** interferes with mitochondrial bioenergetics by disrupting electron transport chain function and ATP synthesis, ultimately promoting mitochondrial dysfunction and cell death [62]. The same compound was also evaluated in HEp-2 (Human Epidermoid Carcinoma) cells, where it exhibited a dose-dependent cytotoxic effect with an IC_50_ value of 20 μM. Although these findings may suggest potential antiproliferative effects, further studies are required to determine the selectivity of this compound toward tumor versus non-tumor cells and to better understand its mechanisms of toxicity.

Additional evidence of cytotoxicity has been reported for metabolites isolated from *Dalea pogonathera*. In this species, flavonoids, flavan-3-ols, and chalcones were evaluated in vitro in hamster splenocytes using flow cytometry assays with Annexin V and propidium iodide staining. Several compounds, particularly diprenylated flavanones and chalcones, produced a marked reduction in cell viability, with some compounds decreasing cell survival by approximately 98%. Similarly, pogonatheridin A, a chalcone derivative, induced a 74.3% reduction in cell viability. Interestingly, the study suggested that the number of prenyl substituents directly influences toxicity, as diprenylated compounds exhibited greater cytotoxicity than monoprenylated derivatives. This effect was attributed to the increased lipophilicity conferred by prenyl groups, which may enhance interactions with cellular membranes and consequently increase cellular toxicity [144].

In vivo toxicological evidence is even more limited. A preliminary acute toxicity study of 8PP administered intraperitoneally to adult albino mice reported LD_50_ values of 357 mg/kg in females and 245 mg/kg in males. These findings provide an initial estimate of acute systemic toxicity but do not address oral safety, repeated-dose exposure, target-organ toxicity, or long-term effects [119]. In addition to experimental studies, some traditional uses of *Dalea* species have also been associated with adverse effects. For example, consumption of the aerial parts of *Dalea bicolor*, including stems, leaves, and flowers, has been reported to induce vomiting, suggesting the presence of metabolites capable of producing gastrointestinal irritant effects [145].

**Figure 8:**
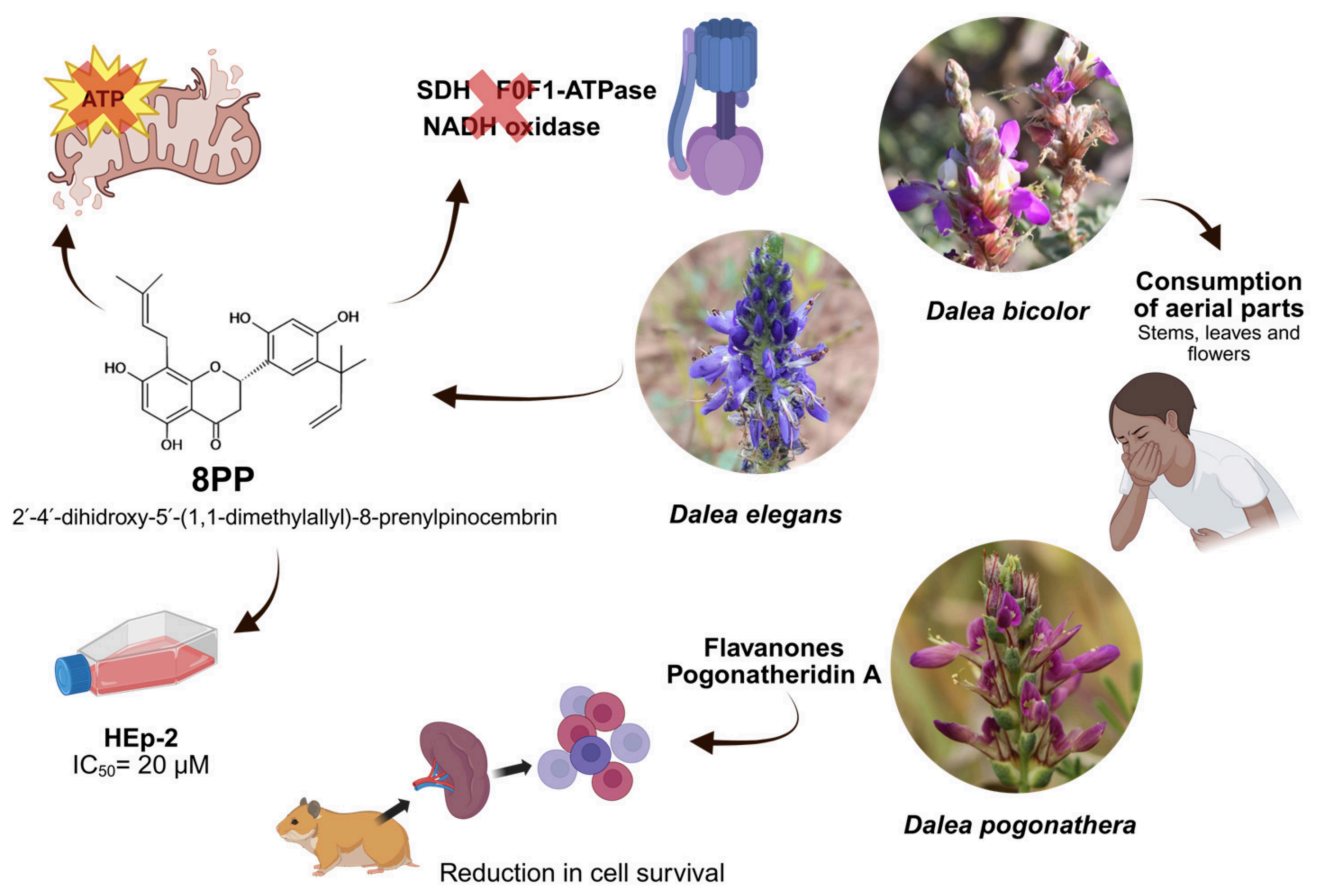
Summary of the main toxicological effects reported on different species of the genus *Dalea* (*D. elegans*, *D. bicolor*, and *D. pogonathera*). Created in BioRender. Biorender, B. (2026) https://.BioRender.com/3zicmla (accessed on 3 June 2025).

Overall, the currently available evidence indicates that species of the genus *Dalea* contain biologically active metabolites capable of exerting both pharmacological and toxicological effects depending on concentration, exposure time, and structural characteristics. In particular, prenylated flavonoids appear to play a dual role, contributing to therapeutic activities while also exhibiting cytotoxic and mitochondrial effects at higher concentrations. Nevertheless, toxicological studies within the genus remain limited and fragmented. Therefore, comprehensive evaluations integrating mechanistic in vitro assays, long-term in vivo studies, pharmacokinetic analyses, and selectivity assessments are still required to establish the safety profile of *Dalea*-derived compounds and support their future pharmacological applications. Figure 8 summarizes the toxicological findings related to the genus *Dalea*.

## 8. Future Perspectives and Research Gaps

Despite the growing interest in the genus *Dalea* as a source of bioactive metabolites, current research remains fragmented and limited to a relatively small number of species. Most phytochemical and pharmacological investigations have focused on prenylated flavonoids, chalcones, rotenoids, and related phenolic compounds; however, the vast majority of *Dalea* species remain chemically unexplored. Given the remarkable structural diversity of the metabolites identified to date, particularly prenylated and geranylated flavonoids, the genus represents a largely under investigated reservoir of bioactive specialized metabolites with considerable pharmaceutical potential.

One of the main limitations in current research is the absence of comprehensive metabolomic and chemotaxonomic studies across the genus. Advanced analytical platforms such as LC-MS/MS-based metabolomics, molecular networking, NMR profiling, and dereplication approaches should be implemented to accelerate the identification of novel metabolites and establish phytochemical relationships among species [146,147,148]. Such strategies may also contribute to understanding the ecological and evolutionary significance of prenylated metabolites within arid and semi-arid environments.

Another important challenge involves the variability in metabolite production associated with geographical origin, climatic conditions, soil composition, and environmental stress [37]. Since many *Dalea* species inhabit desert and xerophytic ecosystems, environmental factors such as drought and high ultraviolet radiation likely influence secondary metabolite biosynthesis [149]. Therefore, the development of standardized cultivation, harvesting, and extraction protocols will be essential to ensure reproducibility and consistency in future pharmacological investigations.

Although several studies have demonstrated antibacterial, antifungal, antiparasitic, anti-inflammatory, neuroprotective, anticancer, anti-tyrosinase, and xanthine-oxidase-inhibitory activities, most available evidence remains restricted to in vitro or preliminary in vivo models. In particular, the molecular mechanisms underlying many of these biological effects are still poorly understood. Therefore, future research on the genus *Dalea* should incorporate emerging technologies that are transforming natural product discovery. Untargeted metabolomics combined with high-resolution LC-MS/MS could facilitate the comprehensive characterization of secondary metabolites across *Dalea* species, accelerating the discovery of novel bioactive compounds. Metabolomics also supports compound identification, dereplication, and the association of chemical profiles with biological activities, particularly when integrated with high-throughput screening approaches [140,150]. Furthermore, integrating metabolomics with other omics approaches, including genomics, transcriptomics, and proteomics, together with molecular docking and signaling pathway analyses, could improve the elucidation of biosynthetic pathways, identify pharmacological targets, clarify SAR of prenylated flavonoids and other *Dalea*-derived metabolites, and reveal previously unexplored bioactive compounds [151,152].

Artificial intelligence (AI) also offers promising opportunities for accelerating phytochemical research by improving metabolite annotation, predicting biological targets, and prioritizing compounds for experimental validation. In parallel, advances in synthetic biology and metabolic engineering could provide sustainable strategies for producing valuable *Dalea* metabolites through heterologous expression systems, reducing dependence on wild plant populations [153,154].

Biotechnological approaches also represent a promising research area for the genus. Since many prenylated and geranylated flavonoids occur in low concentrations under natural conditions [155], alternative production systems such as hairy root cultures, elicitation strategies, and plant tissue culture technologies may provide sustainable methods for obtaining larger quantities of rare bioactive compounds [156]. These approaches could significantly facilitate pharmacological studies and future drug development efforts.

Another major limitation is the scarcity of toxicological and pharmacokinetic data. While some studies have reported mitochondrial toxicity and cytotoxic effects associated with highly lipophilic prenylated flavonoids, comprehensive safety evaluations remain largely unavailable. Long-term toxicity, genotoxicity, reproductive toxicity, and pharmacokinetic studies are needed to establish the therapeutic safety of *Dalea*-derived compounds [157].

Finally, conservation and sustainable use should also be considered in future research involving *Dalea* species, particularly those endemic to arid and semi-arid ecosystems of Mexico and South America. Increased phytochemical and pharmacological interest in the genus may eventually lead to overharvesting of wild populations. Therefore, sustainable management strategies, cultivation programs, and conservation-oriented approaches will be necessary to preserve the biodiversity and ecological importance of these species while supporting their potential pharmaceutical applications.

## 9. Conclusions

The genus *Dalea* is an important source of structurally diverse secondary metabolites, including prenylated flavonoids, isoflavonoids, rotenoids, chalcones, stilbenes, and terpenoid-rich essential oils. Among these, prenylated and geranylated flavonoids constitute one of the most distinctive phytochemical features of the genus and account for many of its reported biological activities. Accordingly, this review places particular emphasis on this metabolite class because it represents the most extensively investigated group within *Dalea*, exhibiting the greatest structural diversity and the broadest range of pharmacological activities reported to date. In contrast, other phytochemical classes remain comparatively underexplored due to the limited number of species and studies available rather than their biological relevance, highlighting important opportunities for future phytochemical and pharmacological research.

Current evidence indicates that *Dalea*-derived metabolites exhibit a broad range of biological activities, including antibacterial, antifungal, antiparasitic, anti-inflammatory, neuroprotective, antiproliferative, tyrosinase-inhibitory, and xanthine-oxidase-inhibitory effects. In several cases, these activities appear to be influenced by structural features such as prenylation patterns, hydroxylation profiles, and chromene formation, although the contribution of these structural modifications varies among compounds and biological models.

Despite these findings, pharmacological research on *Dalea* remains limited to a relatively small number of species and relies predominantly on in vitro studies, with comparatively little in vivo evidence and no clinical validation. Future research should therefore prioritize metabolomic-guided characterization of understudied species and metabolite classes, standardized pharmacological and toxicological evaluations, mechanistic studies, pharmacokinetic characterization, and well-designed in vivo models. Such studies will be essential to determine the biological relevance, safety, and translational potential of *Dalea*-derived metabolites.

## Figures and Tables

**Table 1:** Taxonomic classification of the genus *Dalea* L. [23].

Rank	Taxon
Kingdom	Plantae
Clade	Angiosperms
Clade	Eudicots
Clade	Rosids
Order	Fabales
Family	Fabaceae
Subfamily	Papilionoideae
Tribe	Amorpheae
Genus	*Dalea* L.

**Table 2:** Major bioactive compounds isolated from *Dalea* species and their reported pharmacological activities.

Species	Compound	Chemical Class	Reported Activity	Biological Target/Mechanism of Action	Level of Evidence *
*D. elegans*	8PP **(2)**	Prenylated flavanone	Antifungal/Neuroprotective/Dermatological potential	Efflux pump inhibition; ATPase modulation; biofilm disruption [66,118,119,120,121]/Inhibition of GSK-3β [64]/Tyrosinase inhibition [16,129,130]	In silico model In chemico test In vitro test
*D. elegans*	(2′,4′-dihydroxy-5′-(1-dimethylallyl)-6-prenylpinocembrin) (6PP)	Prenylated flavanone	Antifungal	Activity against azole-resistant *Candida albicans*. Enhance the efficacy of antifungal drugs like fluconazole [66]	In vitro test
*D. frutescens*	Sanjuanolide **(12)**	Prenylated chalcone	Anticancer/Antiinflammatory	Mitotic spindle disruption [68]/Cytokine inhibition [96]	In vitro test
*D. purpurea*	Malheuran A **(3)**	Geranylated flavanone	Antibacterial/Anti-inflammatory	Activity against MRSA and VRE/LPS-stimulated RAW264.7 macrophages (NO inhibition [14]	In vitro test
*D. purpurea*	Condensed tannins	Proanthocyanidins	Antibacterial	Activity against *E. coli* [113,114]	In vitro test
*D. purpurea*	Pawhuskin A **(14)**	Prenylated stilbene	Analgesic potential	Opioid receptor antagonist [127]	In vitro test
*D. boliviana*	(-)-(2S)-5,7,2′-trihydroxy-5′- (1‴,1‴-dimethylallyl)-8-prenylflavanone	Prenylated flavanone	Dermatological potential	Tyrosinase inhibition [63]	In chemico test
*D. formosa*	Sedonan A	Isoflavonoid	Antifungal	Activity against *C. albicans* and *C. glabrata* [41,117]	In vitro test
*D. pazensis*	8PP **(2)**	Prenylated flavanone	Antifungal	*Candida albicans* growth inhibition [43]	In vitro test
*D. scandens* var. *paucifolia*	Prenylated flavonoids	Prenylated flavonoids	Antibacterial	Activity against *Staphylococcus aureus* [67]	In vitro test
*D. versicolor*	Prenylated chalcone and stilbenes	Chalcone/stilbenes	Antibacterial	NorA efflux pump inhibition [97]	In vitro test
*D. nana*	Verdean A **(8)**	Prenylated isoflavan	Antibacterial/Antifungal	Activity against MRSA [90]/*Cryptococcus neoformans* and *Candida albicans* [90]	In vitro test
*D. ornata*	Deguelin **(10)**	Rotenoid	Antiparasitic	Activity against *Ancylostoma ceylanicum* [40]	Ex vivo test
*D. ornata* and *D. searlsiae*	Tephrosin **(11)**	Rotenoid	Antiparasitic/antiinsectan	Helminthic [40]/Insect growth inhibition [30]	Ex vivo test In vivo test
*D. searlsiae*	Malheurans A-D **(3)**	Prenylated flavanones	Antibacterial/antiinsectan	Gram-positive bacteria [30]/Insect growth inhibition [30]	In vitro test In vivo test
*D. albiflora*	Albiflorans A-C **(4)**	Prenylated flavanones	Antibacterial/Antifungal	Activity against MRSA and VRE/*Cryptococcus neoformans* [115]	In vitro test
*D. lanata*	Lanatans A-C **(5)**	Prenylated flavanones	Antibacterial/Antifungal	Activity against MRSA and VRE/*Cryptococcus neoformans* [116]	In vitro test

* Classification according to OECD Guidance related to Integrated Approaches to Testing and Assessment [134].

## Data Availability

No new data were created or analyzed in this study. Data sharing is not applicable to this article.

## Supplementary Materials

The following supporting information can be downloaded at: https://www.mdpi.com/article/10.3390/ph19081294/s1, S1: Interactive map of global *Dalea* occurrence records; S2: Instructions for use; S3: Methodology, source code, and usage instructions of Figure 2.

## Abbreviations

The following abbreviations are used in this manuscript:

6PP	2′,4′-dihydroxy-5′-(1-dimethylallyl)-6-prenylpinocembrin
8-PP	2′,4′-dihydroxy-5′-(1‴,1‴-dimethylallyl)-8-prenylpinocembrin
SAR	Structure-Activity Relationships
AChE	Acetylcholinesterase
ATP	adenosine triphosphate
CDR	*Candida* Drug Resistance
COX-2	cyclooxygenase-2
DMAPP	dimethylallyl diphosphate
EC	Effective Concentration
FICI	Fractional Inhibitory Concentration Index
FPP	farnesyl diphosphate
GPP	geranyl diphosphate
GSK-3β	glycogen synthase kinase-3β
IC_50_	Half-maximal inhibitory concentration
ICAM-1	Intercellular adhesion molecule-1
IL-1β	interleukin-1 beta
IL-6	interleukin-6
LC-MS	Liquid chromatography-mass spectrometry
LD_50_	Lethal Dose 50%
LPS	Lipopolysaccharide
MACP-1	mitochondrial anion carrier proteins-1
MEP	methylerythritol phosphate
MIC	minimum inhibitory concentration
MRSA	methicillin-resistant *S. aureus*
MVA	Mevalonate
NADH	reduced nicotinamide adenine dinucleotide
NDs	neurodegenerative diseases
NGF	nerve growth factor
NMR	Nuclear Magnetic Resonance
P-gp	P-glycoprotein
SDH	succinate dehydrogenase
SMIC_80_	Sessile Minimum Inhibitory Concentration 80%
TNF-α	tumor necrosis factor-α
VRE	vancomycin-resistant enterococci
XO	xanthine oxidase
8PG	8-prenylgenistein
