# Supplementary material for: Phytochemistry and Pharmacological Potential of the Genus Dalea: Current Evidence and Future Directions
Source: Pharmaceuticals (Basel). 2026 Aug 15;19(8):1294. doi: 10.3390/ph19081294 (PMC13516910; doi:10.3390/ph19081294)
Supplement: Supplementary file 1 [file pharmaceuticals-19-01294-s001.zip › pharmaceuticals-4405494-supplementary.pdf]

Supplementary Material

S1: Interactive map of global Dalea occurrence records

An interactive Leaflet map displaying global occurrence records of the genus *Dalea* is available at:

Interactive map

https://lizethmuzquiz.github.io/Dalea_GBIF_Resources/output/Dalea_interactive_map.html

The complete source code, supplementary documentation, and resources required to reproduce the interactive map are available in the GitHub repository:

GitHub repository

https://github.com/lizethmuzquiz/Dalea_GBIF_Resources

The repository includes:

- R script used to generate the interactive map.
- Interactive HTML map.
- Supplementary documentation.
- README describing the repository contents.
- License information.

S2: Instructions for use

The interactive map displays global occurrence records of the genus *Dalea* retrieved from the Global Biodiversity Information Facility (GBIF).

Three selectable basemaps are available (CartoDB Positron, OpenStreetMap, and Esri World Imagery), allowing users to visualize occurrence records under different cartographic backgrounds.

Occurrence records are represented by blue circular markers. When multiple records occur in close geographic proximity, they are automatically grouped into numbered clusters to improve visualization of dense areas. The number displayed within each cluster indicates the total number of occurrence records represented. As users zoom in, clusters progressively separate until individual occurrence records become visible.

Selecting an individual marker opens a popup displaying:

- Accepted species name
- GBIF scientific name
- Original scientific name
- Collector
- Country
- State or province
- Locality
- Collection year

Users can freely zoom, pan, switch between basemaps, and explore occurrence records worldwide.

S3: Methodology

Data acquisition

Occurrence records of the genus *Dalea* were obtained from the Global Biodiversity Information Facility (GBIF) on June 3, 2025. The downloaded dataset comprised 104,234 occurrence records and included taxonomic information, collection metadata, and geographic coordinates.

Data filtering

Data processing was performed in R version 4.5.1.

Records were filtered to retain only observations identified at the species level (taxonRank = "SPECIES") and containing valid geographic coordinates (non-missing latitude and longitude values). The standardized GBIF accepted species name contained in the species field was used throughout the analysis.

Geographic data cleaning

Geographic coordinates were cleaned using the CoordinateCleaner package by applying the following quality-control tests:

- capitals
- centroids
- equal coordinates
- GBIF headquarters
- biodiversity institutions
- seas
- zero coordinates

Only records that passed all quality-control tests were retained.

Preparation of the visualization dataset

To improve map readability and rendering performance, duplicate occurrence records sharing the same accepted species name and identical geographic coordinates were removed only from the visualization dataset. The cleaned occurrence dataset itself was otherwise preserved.

Country names were generated from ISO 3166-1 alpha-2 country codes using the countrycode package.

Following data cleaning, the visualization dataset contained 43,857 occurrence records representing 175 accepted species.

Interactive map generation

The interactive map was generated using the leaflet package and includes three selectable basemaps (CartoDB Positron, OpenStreetMap, and Esri World Imagery).

Occurrence records were represented as blue circular markers. Marker clustering was implemented to automatically aggregate nearby records at lower zoom levels and progressively separate them into individual occurrences as users zoom in, thereby improving visualization of large datasets.

Selecting an individual marker displays a popup containing the accepted species name, GBIF scientific name, original scientific name, collector, country, state or province, locality, and collection year.

The map also includes a scale bar and a summary panel reporting the number of occurrence records, accepted species, data source, and download date.

Reproducibility

The complete R script used to generate the interactive map, together with the interactive HTML file and supporting documentation, is freely available through the GitHub repository listed above.

The original occurrence records can be obtained directly from GBIF using the download described in these Supplementary Methods.
